# Supplementary material for: VERSO: A comprehensive framework for the inference of robust phylogenies and the quantification of intra-host genomic diversity of viral samples
Source: Patterns (N Y). 2021 Jan 28;2(3):100212. doi: 10.1016/j.patter.2021.100212 (PMC7953447; doi:10.1016/j.patter.2021.100212)
Supplement: Document S2. Article plus supplemental information [file mmc5.pdf]

Patterns

Article

VERSO: A comprehensive framework for the inference of robust phylogenies and the quantification of intra-host genomic diversity of viral samples

Graphical abstract

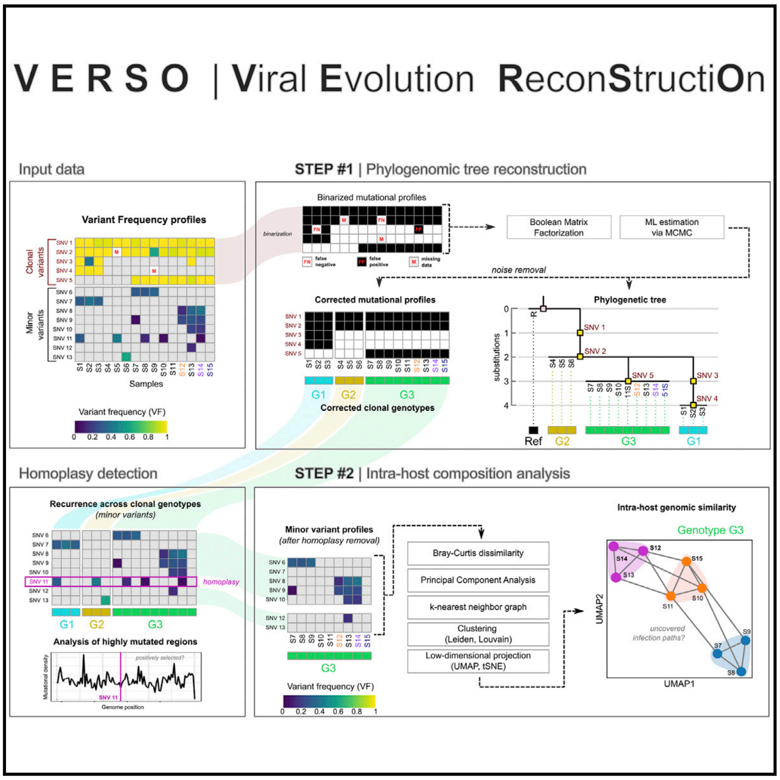

Highlights

- The analysis of raw sequencing data improves the reconstruction of viral evolution
- Our method reconstructs robust phylogenies with noisy data and sampling limitations
- The dissection of intra-host genomic diversity reveals undetected infection chains
- The identification of positively selected variants may drive experimental research

Authors

Daniele Ramazzotti, Fabrizio Angaroni, Davide Maspero, Carlo Gambacorti-Passerini, Marco Antoniotti, Alex Graudenzi, Rocco Piazza

Correspondence

alex.graudenzi@ibfm.cnr.it (A.G.),
rocco.piazza@unimib.it (R.P.)

In Brief

The generation of reliable phylogenomic models describing the evolution of SARS-CoV-2 is essential to explain its diffusion and to possibly predict the next evolutionary steps. We introduce a data-science framework that is an improvement on existing methods, by accounting for noise and sampling limitations in sequencing data and by dissecting the intra-host diversity of single samples. The application to large-scale datasets demonstrates that our approach can improve the estimation of SARS-CoV-2 evolution, refine contact tracing, and pinpoint possibly hazardous mutations.

Ramazzotti et al., 2021, Patterns 2, 100212
March 12, 2021 © 2021 The Author(s).
https://doi.org/10.1016/j.patter.2021.100212

Patterns

CellPress
OPEN ACCESS

Article

VERSO: A comprehensive framework for the inference of robust phylogenies and the quantification of intra-host genomic diversity of viral samples

Daniele Ramazzotti,[1] Fabrizio Angaroni,[2] Davide Maspero,[2,3] Carlo Gambacorti-Passerini,[1] Marco Antoniotti,[2,4] Alex Graudenzi,[3,4,5,6,*] and Rocco Piazza[1,5,*]

[1]Department of Medicine and Surgery, Università degli Studi di Milano-Bicocca, Monza, Italy
[2]Department of Informatics, Systems and Communication, Università degli Studi di Milano-Bicocca, Milan, Italy
[3]Inst. of Molecular Bioimaging and Physiology, Consiglio Nazionale delle Ricerche (IBFM-CNR), Segrate, Milan, Italy
[4]Bicocca Bioinformatics, Biostatistics and Bioimaging Centre – B4, Milan, Italy
[5]Senior author
[6]Lead contact
*Correspondence: alex.graudenzi@ibfm.cnr.it (A.G.), rocco.piazza@unimib.it (R.P.)
https://doi.org/10.1016/j.patter.2021.100212

THE BIGGER PICTURE The gravity of the COVID-19 pandemic has fostered a surge of works analyzing SARS-CoV-2 consensus sequences to reconstruct phylogenomic models of its evolution and diffusion. Yet, such approaches do not account for intra-host genomic diversity and may deliver inaccurate predictions in conditions of noisy data and sampling limitations.

We propose VERSO, a data-science framework for the characterization of viral evolution from sequencing data. By accounting for uncertainty in the data, VERSO delivers robust phylogenies also in conditions of limited sampling and noisy observations. Additionally, the in-depth characterization of the intra-host genomic diversity of samples allows one to identify undetected infection chains and clusters and to intercept variants possibly undergoing positive selection. Accordingly, the joint application of our method and data-driven epidemiological models may deliver a high-precision platform for contact tracing and pathogen surveillance and characterization.

1 2 3 4 5 **Proof-of-Concept:** Data science output has been formulated, implemented, and tested for one domain/problem

SUMMARY

We introduce VERSO, a two-step framework for the characterization of viral evolution from sequencing data of viral genomes, which is an improvement on phylogenomic approaches for consensus sequences. VERSO exploits an efficient algorithmic strategy to return robust phylogenies from clonal variant profiles, also in conditions of sampling limitations. It then leverages variant frequency patterns to characterize the intra-host genomic diversity of samples, revealing undetected infection chains and pinpointing variants likely involved in homoplasies. On simulations, VERSO outperforms state-of-the-art tools for phylogenetic inference. Notably, the application to 6,726 amplicon and RNA sequencing samples refines the estimation of severe acute respiratory syndrome coronavirus 2 (SARS-CoV-2) evolution, while co-occurrence patterns of minor variants unveil undetected infection paths, which are validated with contact tracing data. Finally, the analysis of SARS-CoV-2 mutational landscape uncovers a temporal increase of overall genomic diversity and highlights variants transiting from minor to clonal state and homoplastic variants, some of which fall on the spike gene. Available at: https://github.com/BIMIB-DISCo/VERSO.

INTRODUCTION

The outbreak of coronavirus disease 2019 (COVID-19), which started in late 2019 in Wuhan (China)[1,2] and was declared a pandemic by the World Health Organization, is fueling the publication of an increasing number of studies aimed at exploiting the information provided by the viral genome of severe acute respiratory syndrome-coronavirus 2 (SARS-CoV-2) virus to identify its

This is an open access article under the CC BY license (http://creativecommons.org/licenses/by/4.0/).

proximal origin, characterize the mode and timing of its evolution, as well as to define descriptive and predictive models of geographical spread and evaluate the related clinical impact.[3–5] As a matter of fact, the mutations that rapidly accumulate in the viral genome[6] can be used to track the evolution of the virus and, accordingly, unravel the viral infection network.[7,8]

At the time of this writing, numerous independent laboratories around the world are isolating and sequencing SARS-CoV-2 samples and depositing them on public databases (e.g., GISAID[9]) whose data are accessible via the Nextstrain portal.[10] Such data can be employed to estimate models from genomic epidemiology and may serve, for instance, to estimate the proportion of undetected infected people by uncovering cryptic transmissions, as well as to predict likely trends in the number of infected, hospitalized, dead, and recovered people.[11–13]

More in detail, most studies employ phylogenomic approaches that process consensus sequences, which represent the dominant virus lineage within each infected host. A growing plethora of methods for phylogenomic reconstruction is available to this end, all relying on different algorithmic frameworks, including distance-matrix, maximum parsimony, maximum likelihood, or Bayesian inference, with various substitution models and distinct evolutionary assumptions (see, e.g., Refs.[10,14–22]). However, while such methods have repeatedly proven effective in unraveling the main patterns of evolution of viral genomes with respect to many different diseases, including SARS-CoV-2,[10,23–25] at least two issues can be raised.

First, most phylogenomics methods might produce unreliable results when dealing with noisy data, for instance due to sequencing issues, or with data collected with significant sampling limitations,[14,26,27] as witnessed for most countries during the epidemics.[28,29]

Second, most methods do not consider the key information on intra-host minor variants (also referred to as minority variants or intra-host single nucleotide variants), which can be retrieved from whole-genome deep sequencing raw data and might be essential to improve the characterization of the infection dynamics and to pinpoint positively selected variants.[30–32] Due to the high replication, mutation, and recombination rates of RNA viruses, subpopulations of mutant viruses, also known as viral quasispecies,[30] typically emerge and coexist within single hosts, and are supposed to underlie most of the adaptive potential to the immune system response and to anti-viral therapies.[31,33,34] In this regard, many recent studies highlighted the noteworthy amount of intra-host genomic diversity in SARS-CoV-2 samples,[35–43] similarly to what has already been observed in many distinct infectious diseases.[8,32,44–48]

Here, we introduce VERSO (viral evolution reconstruction), a new comprehensive framework for the inference of high-resolution models of viral evolution from raw sequencing data of viral genomes (see Figure 1). VERSO includes two consecutive algorithmic steps.

Step #1: robust phylogenomic inference from clonal variant profiles

VERSO first employs a probabilistic noise-tolerant framework to process binarized clonal variant profiles (or, alternatively, consensus sequences), to return a robust phylogenetic model also in conditions of sampling limitations and sequencing issues. By adapting algorithmic strategies widely employed in cancer evolution analysis,[49–52] VERSO is able to correct false-positive and false-negative variants, can manage missing observations due to low coverage, and is designed to group samples with identical (corrected) clonal genotype in polytomies, avoiding ungrounded arbitrary orderings. As a result, the accurate and robust phylogenomic models produced by VERSO may be used to improve the parameter estimation of epidemiological models, which typically rely on limited and inhomogeneous data.[11,29] Notice that this step can be executed independently from step #2; for instance, in case raw sequencing data are not available.

Homoplasy detection (clonal variants)

The first step of VERSO allows one to identify clonal mutations that might be involved in reticulation events[53,54] and, in particular, in homoplasies, possibly due to positive selection in a scenario of convergent/parallel evolution,[55] founder effects,[31] or mutational hotspots.[56] Such information might be useful to drive the design of opportune treatments and vaccines; for instance, by blacklisting positively selected genomic regions.

Step #2: characterization of intra-host genomic diversity

In the second step, VERSO exploits the information on variant frequency (VF) profiles obtained from raw sequencing data (if available), to characterize and visualize the intra-host genomic similarity of hosts with identical (corrected) clonal genotype. In fact, even though the extent and modes of transmission of quasispecies from a host to another during infections are still elusive,[31,57] patterns of co-occurrence of minor variants detected in hosts with identical clonal genotype may provide an indication on the presence of undetected infection paths.[8,58] For this reason, the second step of VERSO is designed to characterize and visualize the genomic similarity of samples by exploiting dimensionality reduction and clustering strategies typically employed in single-cell analyses.[59] Alternative approaches for the analysis of quasispecies, yet with different goals and algorithmic assumptions, have been proposed, for instance in Refs.[60–63] and recently reviewed in Knyazev et al.[64] As specified above, VERSO step #2 is executed on groups of samples with identical clonal genotype: the rationale is that the transmission of minor variants implicates the concurrent transfer of clonal variants, excluding the rare cases in which the VF of a clonal variant significantly decreases in a given host; for instance due to mutation losses (e.g., via recombination-associated deletions or via multiple mutations hitting an already mutated genome location[34]) or to complex horizontal evolution phenomena (e.g., super-infections[65,66]). Conversely, the transmission of clonal variants does not necessarily implicate the transfer of all minor variants, which are affected by complex recombination and transmission effects, such as bottlenecks.[31,57] As a final result, VERSO allows one to visualize the genomic similarity of samples on a low-dimensional space (e.g., UMAP [uniform manifold approximation and projection][67] or tSNE [t-distributed stochastic neighbor embedding][68]) representing the intra-host genomic diversity, and to characterize high-resolution infection chains, thus overcoming the limitations of methods relying on consensus sequences.

Homoplasy detection (minor variants)

Importantly, minor variants observed in hosts with distinct clonal genotypes (identified via VERSO step #1) may indicate

CellPress
OPEN ACCESS

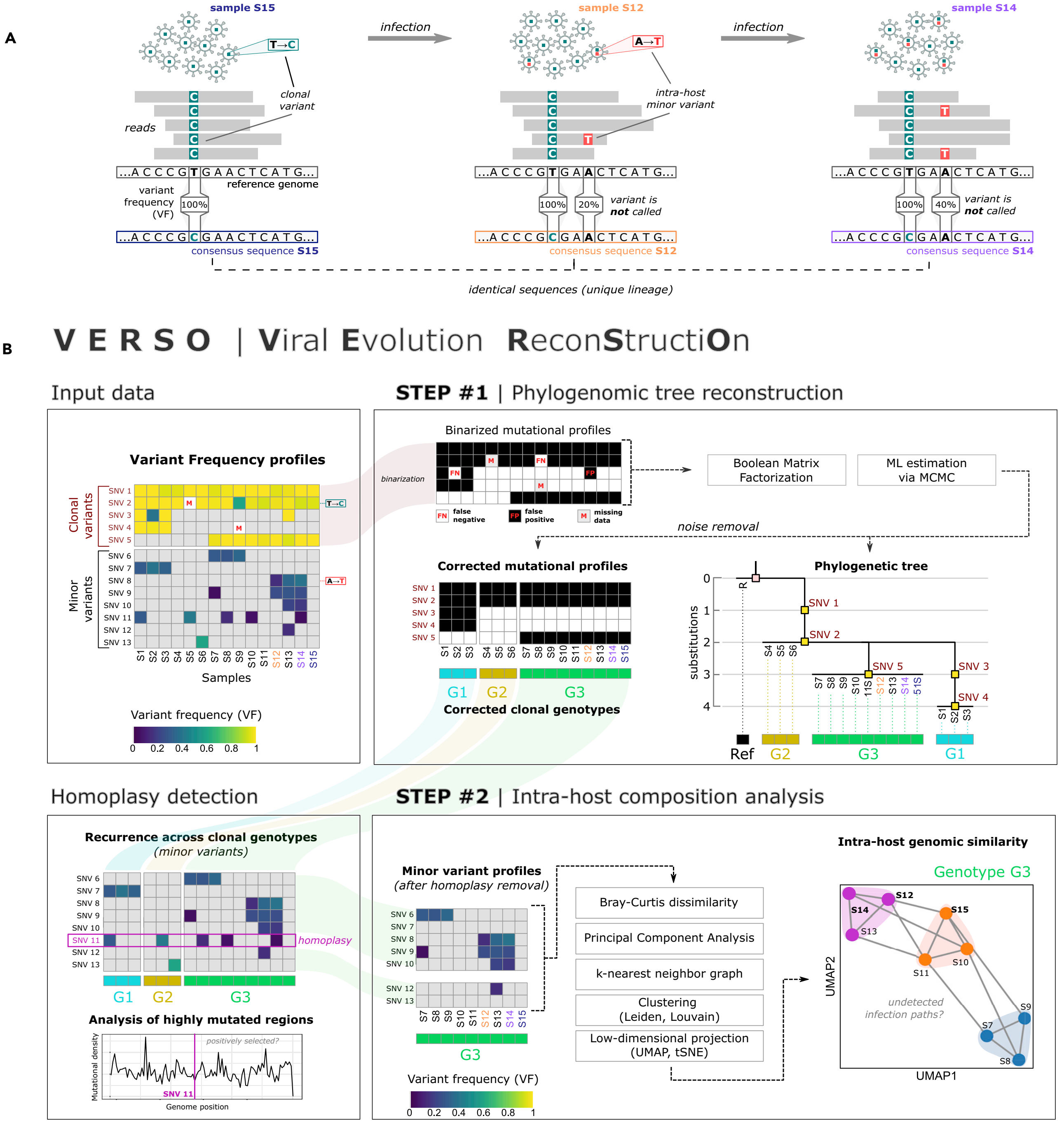

Figure 1. VERSO framework for viral evolution inference and intra-host genomic diversity quantification

(A) In this example, three hosts infected by the same viral lineage are sequenced. All hosts share the same clonal mutation (T>C, green), but two of them (#2 and #3) are characterized by a distinct minor mutation (A>T, red), which randomly emerged in host #2 and was transferred to host #3 during the infection. Standard sequencing experiments return an identical consensus sequence for all samples, by employing a threshold on VF and by selecting mutations characterizing the dominant lineage.

(B) VERSO takes as input the VF profiles of samples, generated from raw sequencing data. In step #1, VERSO processes the binarized profiles of clonal variants and solves a Boolean matrix factorization problem by maximizing a likelihood function via MCMC, in order to correct false-positives/-negatives and missing data. As output, it returns both the corrected mutational profiles of samples and the phylogenetic tree, in which samples with identical corrected clonal genotypes are grouped in polytomies. Corrected clonal genotypes are then employed to identify homoplasies of minor variants, which are further investigated to pinpoint positively selected mutations. The VF profile of minor variants (excluding homoplasies) is processed by step #2 of VERSO, which computes a refined genomic distance among hosts (via Bray-Curtis dissimilarity, after PCA) and performs clustering and dimensionality reduction, in order to project and visualize samples on a 2D space, representing the intra-host genomic diversity and the distance among hosts. This allows one to identify undetected transmission paths among samples with identical clonal genotype.

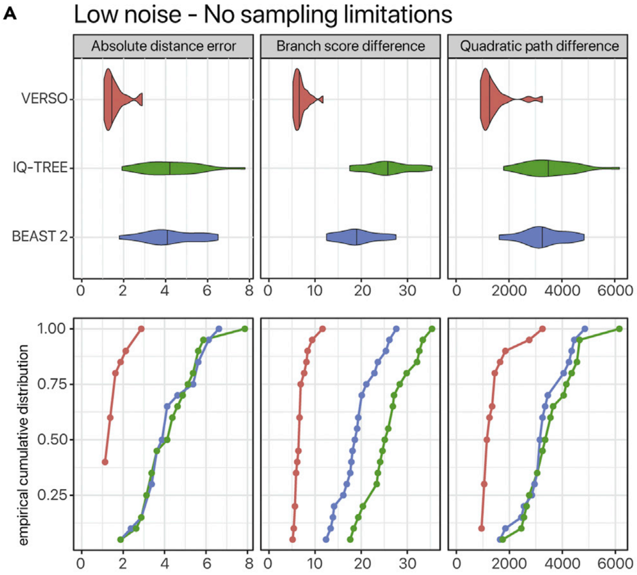

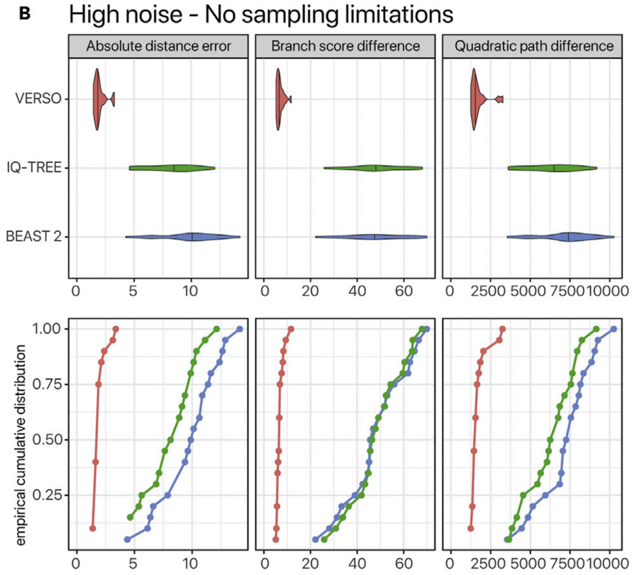

VERSO vs.	Improv. median	U-test 2-sided p-Value	Improv. median	U-test 2-sided p-Value	Improv. median	U-test 2-sided p-Value
IQ-TREE	-67.7%	< .001	-74.3%	< .001	-65.4%	< .001
BEAST 2	-66.2%	< .001	-65.3%	< .001	-62.4%	< .001

20 topologies: n = 1000, α = 0.05, β = 0.05, m ∈ {14,...,31}

VERSO vs.	Improv. median	U-test 2-sided p-Value	Improv. median	U-test 2-sided p-Value	Improv. median	U-test 2-sided p-Value
IQ-TREE	-78.6%	< .001	-86.1%	< .001	-76.6%	< .001
BEAST 2	-81.7%	< .001	-85.8%	< .001	-79.5%	< .001

20 topologies: n = 1000, α = 0.10, β = 0.10, m ∈ {14,...,31}

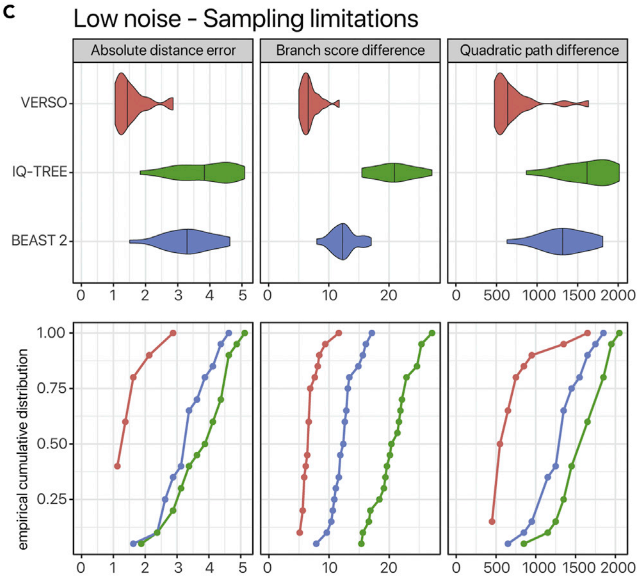

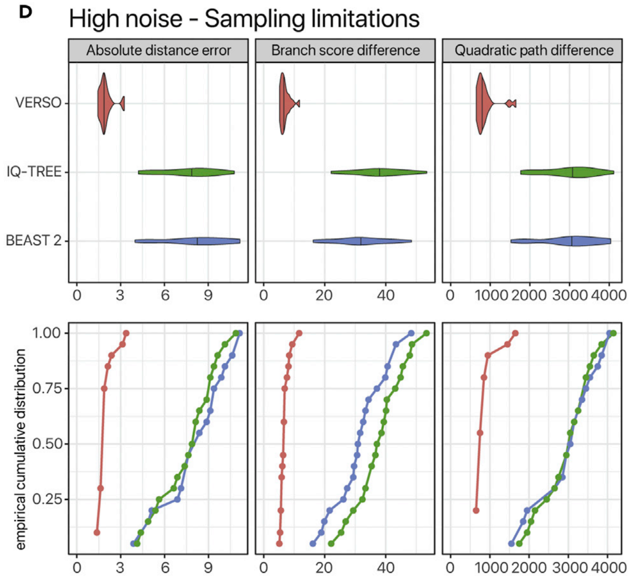

VERSO vs.	Improv. median	U-test 2-sided p-Value	Improv. median	U-test 2-sided p-Value	Improv. median	U-test 2-sided p-Value
IQ-TREE	-66.4%	< .001	-68.5%	< .001	-64.9%	< .001
BEAST 2	-59.8%	< .001	-47.3%	< .001	-56.3%	< .001

20 topologies: n = 500, α = 0.05, β = 0.05, m ∈ {14,...,31}

VERSO vs.	Improv. median	U-test 2-sided p-Value	Improv. median	U-test 2-sided p-Value	Improv. median	U-test 2-sided p-Value
IQ-TREE	-76.6%	< .001	-82.7%	< .001	-74.9%	< .001
BEAST 2	-78.1%	< .001	-79.0%	< .001	-75.3%	< .001

20 topologies: n = 500, α = 0.10, β = 0.10, m ∈ {14,...,31}

VERSO IQ-TREE BEAST 2

Figure 2. Comparative assessment on simulated data

(A–D) Synthetic datasets were generated via the widely used coalescent model simulator msprime[70] (see the Supplementary Material and Table S1 for the parameter settings). Twenty distinct topologies with 1,000 samples were generated, including a number of distinguishable variants in the range (14, 31). For each topology, four synthetic datasets were generated, with different sample sizes (n = 1000, 500), and different combinations of false-positives and false-negatives

(legend continued on next page)

homoplasies, due to mutational hotspots, phantom mutations, or to positive selection.[56] VERSO pinpoints such variants for further investigations and allows one to exclude them from the computation of the VF-based genomic similarity prior to VERSO step #2, to reduce the possible confounding effects.

To summarize, VERSO (1) returns accurate and robust phylogenies of viral samples, by removing noise from clonal variant profiles; (2) detects reticulation events due to homoplasies of clonal variants; (3) exploits minor variant profiles to characterize and visualize the intra-host genomic similarity of samples with identical (corrected) clonal genotype, thus pinpointing undetected infection paths; (4) allows one to identify and characterize homoplastic minor variants, which might be due to positive selection or mutational hotspots.

To assess the accuracy and robustness of the results produced by VERSO, we performed an extensive array of simulations, and compared with two state-of-the-art methods for phylogenetic reconstruction; i.e., IQ-TREE[10] and BEAST 2.[22] As a major result, VERSO outperforms competing methods in all settings and also in condition of high noise and sampling limitations.

Furthermore, we applied VERSO to two large-scale datasets, generated via amplicon and RNA-seq Illumina sequencing protocols, including 3,960 and 2,766 samples, respectively. The robust phylogenomic models delivered via VERSO step #1 allow us to refine the current estimation on SARS-CoV-2 evolution and spread. Besides, thanks to the in-depth analysis of the mutational landscape of both clonal and minor variants, we could identify a number of variants undergoing transition to clonality, as well as several homoplasies, including variants likely undergoing positive selection processes.

Remarkably, the infection chains identified via VERSO step #2, by assessing the intra-host genomic similarity of samples with the same clonal genotype, were validated by employing contact tracing data from Rockett et al.[69] This important result, which could not be achieved by analyzing consensus sequences, proves the effectiveness of employing raw sequencing data to improve the characterization of the transmission dynamics, in particular during the early phase of the outbreak, in which a relatively low diversity of SARS-CoV-2 has been observed at the consensus level.

VERSO is released as free open source tool at this link: https://github.com/BIMIB-DISCo/VERSO.

RESULTS

Comparative assessment on simulations

In order to assess the performance of VERSO and compare it with competing approaches, we executed extensive tests on simulated datasets, generated with the coalescent model simulator msprime.[70] Simulations allow one to compute a number of metrics with respect to the ground truth, which in this case is the phylogeny of samples resulting from a backwards-in-time coalescent simulation.[71] Accordingly, this allows one to evaluate the accuracy and robustness of the results produced by competing methods in a variety of in-silico scenarios.

In detail, we selected 20 simulation scenarios with n = 1,000 samples in which a number of clonal variants (with distinguishable profiles) between 14 and 31 was observed. We then inflated the datasets with false-positives with rate α and false-negatives with rate β, in order to mimic sequencing and coverage issues. Moreover, additional datasets were generated via random subsampling of the original datasets, to model possible sampling limitations and sampling biases. As a result, we investigated four simulations settings: (A) low noise, no subsampling; (B) high noise, no subsampling; (C) low noise, subsampling; and (D) high noise, subsampling (see Experimental procedures and the Supplemental experimental procedures for further details; the complete parameter settings of the simulations are provided in Table S1).

VERSO step #1 was compared with two state-of-the-art phylogenetic methods from consensus sequences: IQ-TREE,[10] the algorithmic strategy included in the Nextstrain-Augur pipeline,[72] and BEAST 2.[22] Consensus sequences to be provided as input to such methods were generated from simulation data by employing the reference genome SARS-CoV-2-ANC (see below).

The performance of methods was assessed by comparing the reconstructed phylogeny with the simulated ground truth, in terms of (1) absolute error evolutionary distance, (2) branch score difference,[73] and (3) quadratic path difference[74] (please refer to the Supplemental experimental procedures for a detailed description of all metrics).

Figure 2 shows the performance distribution of all methods with respect to all simulation settings. Notably, VERSO step #1 outperforms competing methods in all scenarios (Mann-Whitney U test, $p < 0.001$ in all cases), with noteworthy percentage improvements, also in conditions of high noise and sampling limitations. This important result shows that the probabilistic framework that underlies VERSO step #1 can produce more robust and reliable results when processing noisy data, as typically observed in real-world scenarios.

Reference genome

Different reference genomes have been employed in the analysis of SARS-CoV-2 origin and evolution. Two genome sequences from human samples, in particular, were used in early phylogenomic studies, namely sequence EPI_ISL_405839 (ref. #1 in the following) used, e.g., in Bastola et al.[75] and sequence EPI_ISL_402125 (ref. #2) used, e.g., in Andersen et al.[3] Excluding the polyA tails, the two sequences are identical for 29,865 of 29,870 genome positions (99.98%) and differ for only five SNPs at locations 8,782, 9,561, 15,607, 28,144, and 29,095, for which ref. #1 has haplotype TTCCT and ref. #2 has haplotype CCTTC.

In order to define a likely common ancestor for both sequences, we analyzed the Bat-CoV-RaTG13 genome (sequence

([α = 0.05, β = 0.05], [α = 0.10, β = 0.10]), for a total of four configurations (A, B, C, and D) and 80 independent datasets. VERSO step #1 was compared with IQ-TREE[10] and BEAST 2,[22] on (1) absolute error evolutionary distance, (2) branch score difference[73] and (3) quadratic path difference[74] with respect to the ground-truth sample phylogeny provided by msprime (see the Supplemental experimental procedures for the description of the metrics). In the upper panels, distributions are shown as violin plots, whereas lower panels include the empirical cumulative distribution functions. The percentage improvement of VERSO with respect to competing methods is shown on all metrics (computed on median values), in addition to the p value of the two-sided Mann-Whitney U test on distributions, for all settings.

EPI_ISL_402131)[1] and the Pangolin-CoV genome (sequence EPI_ISL_410721),[3,4] which were identified as closely related genomes to SARS-CoV-2.[76] In particular, it was hypothesized that SARS-CoV-2 might be a recombinant of an ancestor of Pangolin-CoV and Bat-CoV-RaTG13,[4,77] whereas more recent findings would suggest that the SARS-CoV-2 lineage is the consequence of a direct or indirect zoonotic jump from bats.[76] Whatever the case, both Bat-CoV-RaTG13 and Pangolin-CoV display haplotype TCTCT at locations 8,782, 9,561, 15,607, 28,144 and 29,095 and, therefore, one can hypothesize that such a haplotype was present in the unknown common ancestor of ref. #1 and #2.

For this reason, we generated an artificial reference genome, named SARS-CoV-2-ANC, which is identical to both ref. #1 and #2 on 29,865 (over 29,870) genome locations, includes the polyA tail of ref. #2 (33 bases), and has haplotype TCTCT at locations 8,782, 9,561, 15,607, 28,144, and 29,095 (see Figure S2 for a depiction of the artificial genome generation). SARS-CoV-2-ANC is a likely common ancestor of both genomes and was used for variant calling in downstream analyses (SARS-CoV-2-ANC is released in FASTA format as Data S1). Notice that VERSO pipeline is flexible and can employ any reference genome.

Application of VERSO to 3,960 samples from amplicon sequencing data (dataset #1)

We retrieved raw Illumina Amplicon sequencing data of 3,960 SARS-CoV-2 samples of dataset #1 and applied VERSO to the mutational profiles of 2,906 samples selected after quality check (mutational profiles were generated by executing variant calling via standard practices; see Experimental procedures for further details). Notice that the analysis of this dataset was performed independently from that of dataset #2 in order to exclude possible sequencing-related artifacts or idiosyncrasies.

VERSO step #1: robust phylogenomic inference from clonal variant profiles

We first applied VERSO step #1 to the mutational profile of the 29 variants detected as clonal (VF > 90%) in at least 3% of the samples, in order to reconstruct a robust phylogenomic tree. The VERSO phylogenetic model is displayed in Figure 3A and highlights the presence of 25 clonal genotypes, obtained by removing noise from data, and that define polytomies including different numbers of samples (see Experimental procedures for further details). The mapping between clonal genotype labels and the lineage dynamic nomenclature proposed by Rambaut et al.[78] was obtained via pangolin 2.0[79] and is provided in Data S3.

More in detail, variant g.29095T>C (*N*, synonymous) is the earliest evolutionary event from reference genome SARS-CoV-2-ANC and is detected in 2,454 samples of the dataset. The related clonal genotype G1, which is characterized by no further mutations, identifies a polytomy including 57 Australian, 15 Chinese, 12 American, and one South-African samples.

Three clades originate from G1: a first clade includes clonal genotypes G2 (six samples) and G3 (103), while a second clade includes clonal genotype G4 (86). Clonal genotypes G1ffiG4 are characterized by the absence of single nucleotide variants (SNVs) g.8782T>C (*ORF1ab*, synonymous) and g.28144C>T (*ORF8*, p.84S>L) and correspond to previously identified type A[24] (also type S[82]), which was hypothesized to be an early SARS-CoV-2 type.

The third clade originating from clonal genotype G1 includes all remaining clonal genotypes (G5-G25) and is characterized by the presence of both SNVs g.8782T>C and g.28144C>T. This specific haplotype corresponds to type B[24] (also type L[82]) and an increase of its prevalence has progressively recorded in the population, as one can see in Figure 3, as opposed to type A (S), which was rarely observed in late samples. In this regard, we note that there are currently insufficient elements to support any epidemiological claim on virulence and pathogenicity of such SARS-CoV-2 types, even if recent evidences would suggest the existence of a low correlation.[83]

Variant g.23403A>G (*S*, p.614D>G) is of particular interest, as proven by the increasing number of related studies.[84–87] Such a variant identifies a large clade including 11 clonal genotypes: G15 (493 samples), G16 (1), G17 (512), G18 (25), G19 (118), G20 (94), G21 (648), G22 (90),G23 (127), G24 (4), and G25 (86), for a total of 2,198 total samples, distributed especially in Australia (971), the United States (841), South Africa (257), and Israel (125). Importantly, a constant increase of the prevalence of the haplotype corresponding to such variant is observed in time (see Figure 3), which might hint at ongoing positive selection processes; e.g., due to increased viral transmission. However, this hypothesis is highly debated[88] and, in order to investigate the possible functional effect of such variant and the related clinical implications, *in vivo* and *in vitro* studies are needed.[87]

By looking more in detail at the geo-temporal localization of samples depicted via Microreact[81] (Figure 3B), one can see that the different clonal genotypes are distributed across the world in distinct complex patterns, suggesting that most countries might have suffered from multiple introductions, especially in the early phases of the epidemics. In particular, samples are distributed in 11 countries, with Australia (1,523 samples), United States (910), South Africa (260), Israel (133), and China (45) representing around 99% of the dataset.

The country displaying the largest number of samples is Australia, with 1,523 samples, distributed in 22 different clonal genotypes. The presence of a number of early clonal genotypes (i.e., G1, G2, G3, G4, and G6) supports the hypothesis of multiple introductions of SARS-CoV-2 in Australia. Interestingly, we note that, from the 16th week on, the composition of the Australian sample group tends to be polarized toward clonal genotypes G17 (108/311 $\approx$ 35%) and G25 (82/311 $\approx$ 26%).

910 samples from the United States are included in the dataset, distributed in 17 different clonal genotypes, with G21 being the most abundant in the population (376/910 $\approx$ 41%). Also in this case, samples collected in the initial weeks belong to the ancestral clades, supporting the hypothesis of multiple introductions. Notably, after the 17th week, all American samples display the haplotype g.23403A>G (*S*,p.614D>G) and we notice an overall decrease in genomic diversity, since the observed clonal genotypes pass from 16 (week interval 9–16, 2020) to 8 (week interval 17–29, 2020). Notice that only 49.1% of the American samples have a collection date.

Two-hundred and sixty samples from South Africa are included in the dataset, which are partitioned in six different clonal genotypes, four of which (G1, G8, G14, and G16) include

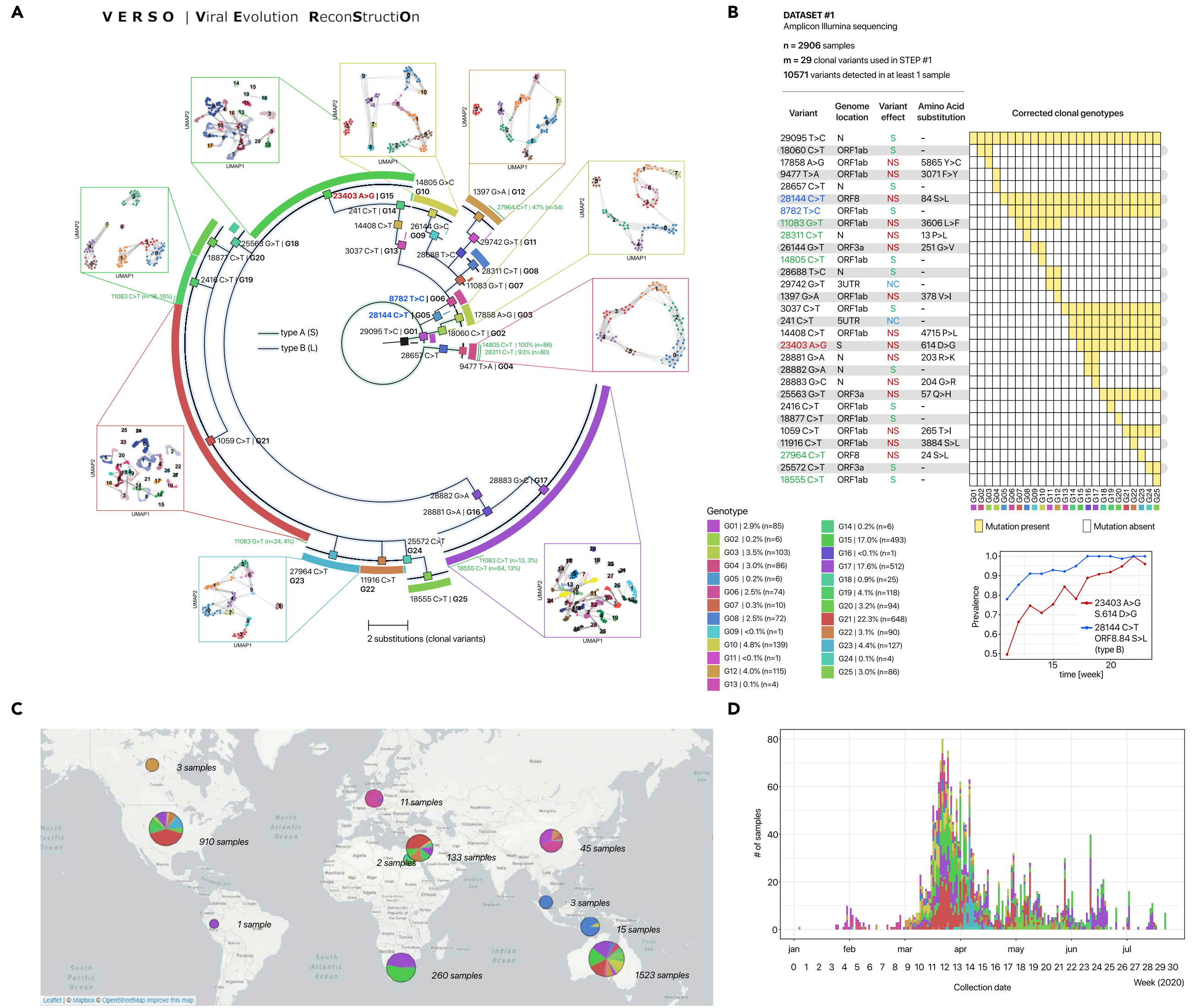

Figure 3. Viral evolution and intra-host genomic characterization of 2906 SARS-CoV-2 samples of via VERSO (dataset #1)

(A) The phylogenetic model returned by VERSO step #1 from the mutational profile of 2,906 samples selected after the quality check, on 29 clonal variants (VF > 90%) detected in at least 3% of the samples of dataset #1 (reference genome: SARS-CoV-2-ANC). Colors mark the 25 distinct clonal genotypes identified by VERSO (the mapping with the lineage nomenclature proposed in Rambaut et al.[78] and generated via pangolin 2.0[79] is provided in File S3). Samples with identical corrected clonal genotypes are grouped in polytomies and the black sample represents the SARS-CoV-2-ANC genome (visualization via FigTree[80]). The green curves juxtaposed to certain polytomies report the number and fraction of samples in which the five homoplastic mutations are observed (only if the mutation is detected in at least 10 samples with the same corrected clonal genotype; see Data S2 for a summary on the samples exhibiting homoplastic clonal variants). The projection of the intra-host genomic diversity computed by VERSO step #2 from VF profiles is shown on the UMAP low-dimensional space for the clonal genotypes including ≥100 samples. Samples are clustered via Leiden algorithm on the kNN graph ($k = 10$), computed on the Bray-Curtis dissimilarity on VF profiles, after PCA. Solid lines represent the edges of the k-NNG.

(B) The composition of the corrected clonal genotypes returned by VERSO step #1 is shown. Clonal SNVs are annotated with mapping on ORFs, synonymous (S), nonsynonymous (NS), and non-coding (NC) states, and related amino acid substitutions. Variants g.8782T>C (*ORF1ab*, synonymous) and g.28144C>T (*ORF8*, p.84S>L) are colored in blue, whereas variant g.23403 A¿G (*S*, p.614 D¿G) is colored in red. The prevalence variation in time of the relative haplotypes (i.e., the fraction of samples displaying such mutations) is also shown. The five homoplastic variants are colored in green.

(C and D) (C) The geo-temporal localization of the clonal genotypes via Microreact[81] and (D) the prevalence variation in time are displayed.

a single sample, whereas 98.46% of the samples exhibit the haplotype g.23403A>G (*S*,p.614D>G) and, specifically, are included in clonal genotypes G15 and G17. Finally, all Chinese samples were collected in the early phase (January–February, 2020) and are characterized by six different clonal genotypes (i.e., G1, G6, G7, G9, G11, and G12).

Homoplasy detection (clonal variants)

Five clonal variants included in our model show apparent violations of the accumulation hypothesis, namely g.11083G>T (*ORF1ab*, p.3606 L>F), g.14805C>T (*ORF1ab*, synonymous), g.18555C>T (*ORF1ab*, synonymous), g.27964C>T (*ORF8*, p.24S>L), and g.28311C>T (*N*,p.13P>L), suggesting that they

might be involved in homoplasies. In Figure 3 the samples in which the five homoplastic variants are detected are highlighted (if the mutation is detected in $\geq$10 samples with the same corrected clonal genotype), whereas in Figure S3 one can find the expanded clonal variant tree, in which the reticulation related to such variants is explicitly depicted.

Some of such variants have been exhaustively studied (e.g., g.11083G>T in van Dorp et al.[89]), specifically to verify possible scenarios of convergent evolution, which may unveil the fingerprint of adaptation of SARS-CoV-2 to human hosts. To this end, particular attention should be devoted to the three non-synonymous substitutions; i.e., g.11083G>T (present in 460 samples, $\approx$16% of the dataset), g.27964C>T (182 samples, $\approx$6%) and g.28311C>T (153 samples, $\approx$5%). As a first result, we note the prevalence dynamics of the haplotypes defined by such variants does not show any apparent growth trend in the population (see Figure S5).

To further investigate if such variants fall in a region prone to mutations of the SARS-CoV-2 genome, we evaluated the mutational density employing a sliding window approach similarly to Soares et al.[90] (see Supplemental experimental procedures for additional details). As shown in Figure S4, the mutational density, computed by considering synonymous minor variants, exhibits a median value of = 0.083 [syn.mutations][nucleotides]$^{-1}$. Interestingly, the three nonsynonymous SNVs (g.11083G>T, g.27964C>T and g.28311C>T) are located within windows with a higher mutational density than the median value: 0.085, 0.124, and 0.1 $\frac{\text{syn.mutations}}{\text{nucleotides}}$, respectively (see Table S5), and this would suggest that they might have originally emerged due to the presence of natural mutational hotspots or phantom mutations.

However, this analysis is not conclusive and further investigations are needed to characterize the functional effect of such mutations and the possible impact in the evolutionary and diffusion process of SARS-CoV-2.

Stability analysis

The choice of an appropriate VF threshold to identify clonal variants and, accordingly, to generate consensus sequences from raw sequencing data might affect the stability of the results of any downstream phylogenomic analysis. On the one hand, loose thresholds might increase the risk of including non-clonal variants in consensus sequences. On the other hand, too strict thresholds might increase the rate of false-negatives, especially with noisy sequencing data.

For this reason, we assessed the robustness of the results produced by VERSO step #1 on dataset #1 when different thresholds in the set $\delta \in \{0.5, 0.6, 0.7, 0.8\}$ are employed to identify clonal variants, with those obtained with default threshold (δ = 0.9), in terms of tree accuracy (see the Supplemental experimental procedures for further details). As one can see in Figure S7, the tree accuracy varies between 0.97 and 0.98 in all settings, proved the results produced by VERSO step #1 are robust with regard to the choice of the VF threshold for clonal variant identification.

VERSO step #2: Characterization of intra-host genomic diversity

We then applied VERSO step #2 to the complete VF profiles of the samples with the same clonal genotype and projected their intra-host genomic diversity on the UMAP low-dimensional space. This was done excluding (1) the clonal variants employed in the phylogenetic inference via VERSO step #1, (2) all minor variants (VF $\leq$ 90%) observed in more than one clonal genotype (i.e., homoplasies) and that are likely emerged independently within the hosts, due to mutational hotspots, phantom mutations, or positive selection (see Experimental procedures and the next subsections). Even though, as expected, the VF profiles of minor variants are noisy, a complex intra-host genomic architecture is observed in several individuals. Moreover, patterns of co-occurrence of minor variants across samples support the hypothesis of transmission from one host to another.

In Figure 3 we display the UMAP plots for the clonal genotypes including more than 100 samples, plus clonal genotype G4 (n = 86 samples), which was used for contact tracing analyses. Such maps describe likely transmission paths among hosts characterized by the same (corrected) clonal genotype and, in most cases, suggests the existence of several distinct infection clusters with different size and density. This result was achieved by exploiting the different properties of clonal and minor variants via the two-step procedure of VERSO.

Contact tracing

To corroborate our findings, we employed the contact tracing data from Rockett et al.,[69] in which 65 samples from dataset #1 are characterized with respect to household, work location, or other direct contacts. Four distinct contact groups, including 36, 15, 12, and 2 samples, respectively, are associated directly or indirectly to three different New South Wales institutions (i.e., institutions #1, #2, and #3) and to the same household environment (household #1).

As a first result, all samples belonging to a specific contact group are characterized by the same clonal genotype, determined via VERSO step #1, a result that confirms recent findings.[42,69] More importantly, the analysis of the intra-host genomic diversity via VERSO step #2 allows one to highly refine this analysis.

In Figure 4 one can find the UMAP plot of clonal genotypes G4, G12, and G21, which include 36 (over 86), 14 (over 115), and 14 (over 648) samples with contact information. Strikingly, the distribution of the pairwise intra-host genomic distance among samples from the same institution/household (computed on the K-nearest neighbor graph [k-NNG] via Bray-Curtis dissimilarity, after principal component analysis [PCA]; see Experimental procedures) is significantly lower with respect to the distance of all samples with the same clonal genotype (p value of the Mann-Whitney U test <0.001 in all cases). Furthermore, all samples belonging to the same contact group are connected in the k-NNG, while a noteworthy proportion of samples without contact information in genotypes G12 and G21 are placed in disconnected graphs (24.9% and 76.4%, respectively).

This major result suggests that patterns of co-occurrence of minor variants can indeed provide useful indication on contact tracing dynamics, which would be masked when employing consensus sequencing data. Accordingly, the algorithmic strategy employed by VERSO step #2 and, especially, the identification of the k-NNG on intra-host genomic similarity provides an effective tool to dissect the complexity of viral evolution and transmission, which might in turn improve the reliability of currently available contact tracing tools.

Homoplasy detection (minor variants)

Several minor variants are found in samples with distinct clonal genotypes and might indicate the presence of homoplasies. In

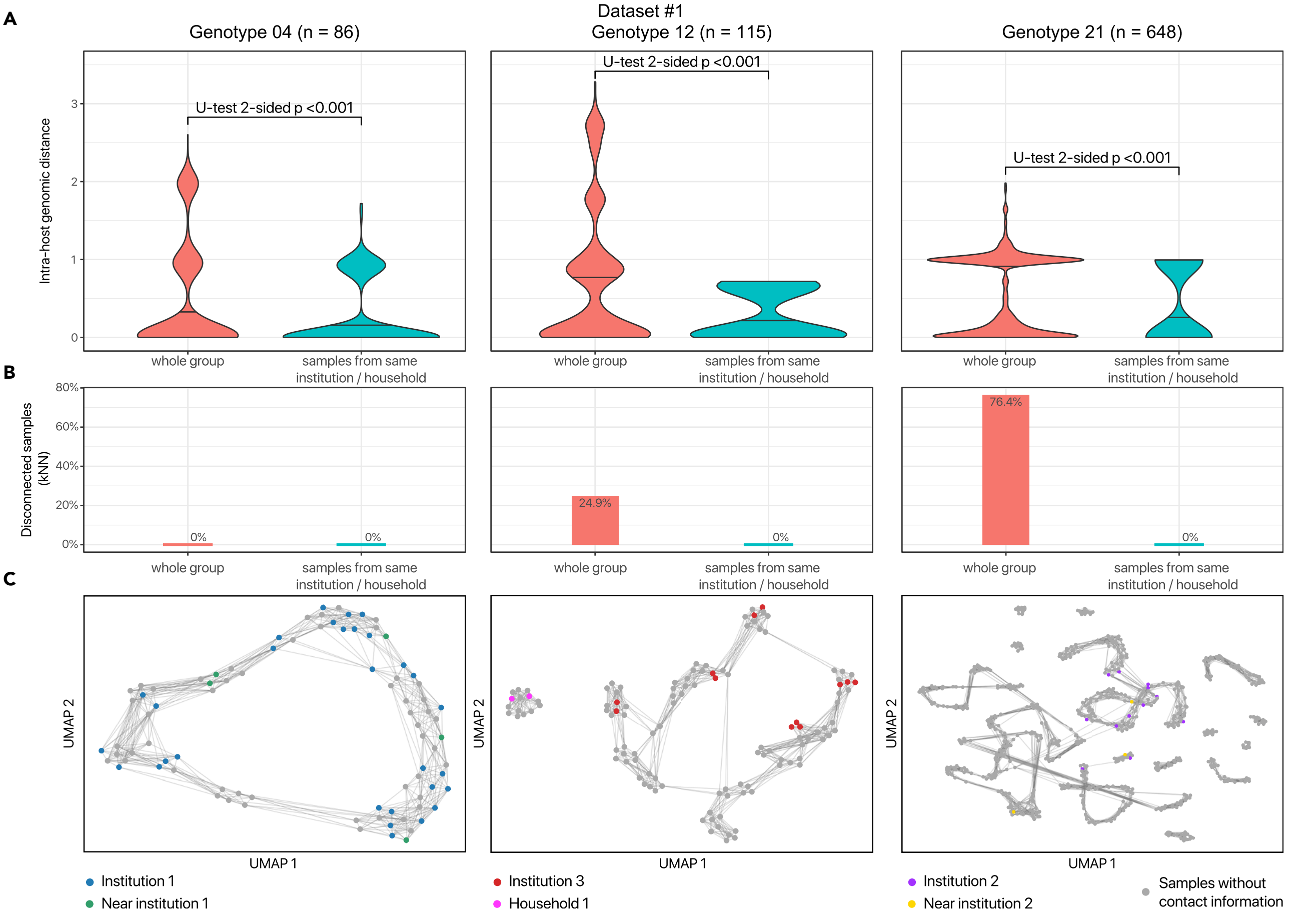

Figure 4. Infection dynamics revealed via characterization of intra-host genomic similarity (dataset #1)

(A) The distribution of the pairwise intra-host genomic distance (computed via Bray-Curtis dissimilarity on the kNN graph, with $k = 10$, after PCA; see Experimental procedures) for the samples belonging to the same household or institution (including samples marked as near), versus the pairwise distance of all samples belonging to clonal genotypes G4, G12, and G21. The p values of the Mann-Whitney U test two-sided are also shown.

(B) The proportion of samples that are disconnected in the kNN graph, with respect to the samples belonging to the same household or institution (including samples marked as near) and with respect to all samples.

(C) The UMAP projection of the intra-host genomic diversity of the samples belonging to clonal genotypes G4, G12, and G21, returned by VERSO step #2.

this respect, the heatmap in Figure 5F returns the distribution of minor SNVs with respect to (1) the number of distinct clonal genotypes in which they are detected, and (2) the mutational density of the region in which they are located (see the Supplemental experimental procedures for details on the mutational density analysis).

The intuition is that the variants detected in single clonal genotypes (left region of the heatmap) are likely spontaneously emerged private mutations, or the result of infection events between hosts with same clonal genotype (see above). Conversely, SNVs found in multiple clonal genotypes (right region of the heatmap) may have emerged due to positive selection in a parallel/convergent evolution scenario, or to mutational hotspots or phantom mutations. To this end, the mutational density analysis provides useful information to pinpoint mutation-prone regions of the genome.

Interestingly, a significant number of minor variants are observed in multiple clonal genotypes and fall in scarcely mutated regions of the genome (see Figure S4). This would suggest that some of these variants might have been positively selected, due to some possible functional advantage or to transmission-related founder effects. In this respect, we further focused our investigation on a list of 80 candidate minor variants that (1) are detected in more than one clonal genotype, (2) are present in at least 10 samples, (3) are nonsynonymous, and (4) fall in a region of the genome with mutational density lower than the median value (see Table S6 for details on such variants). In the following, we focus on a subset of such variants falling on the spike gene of the SARS-CoV-2 genome.

Considerations on homoplasies falling on the spike gene

The spike protein of SARS-CoV-2 plays a critical role in the recognition of the ACE2 receptor and in the ensuing cell membrane fusion process.[91] We prioritized three candidate homoplastic minor variants occurring on the SARS-CoV-2 spike gene (S) (see Table S6). Interestingly, two out of three, namely g.24552T>C (p.997I>T) and g.24557G>T (p.999G>C), detected in 57 samples in total (10 and 47 samples, respectively), clustered in the so-called connector region (CR), bridging between the two heptad repeat regions (HR1 and HR2) of the S2 subunit of the spike protein.

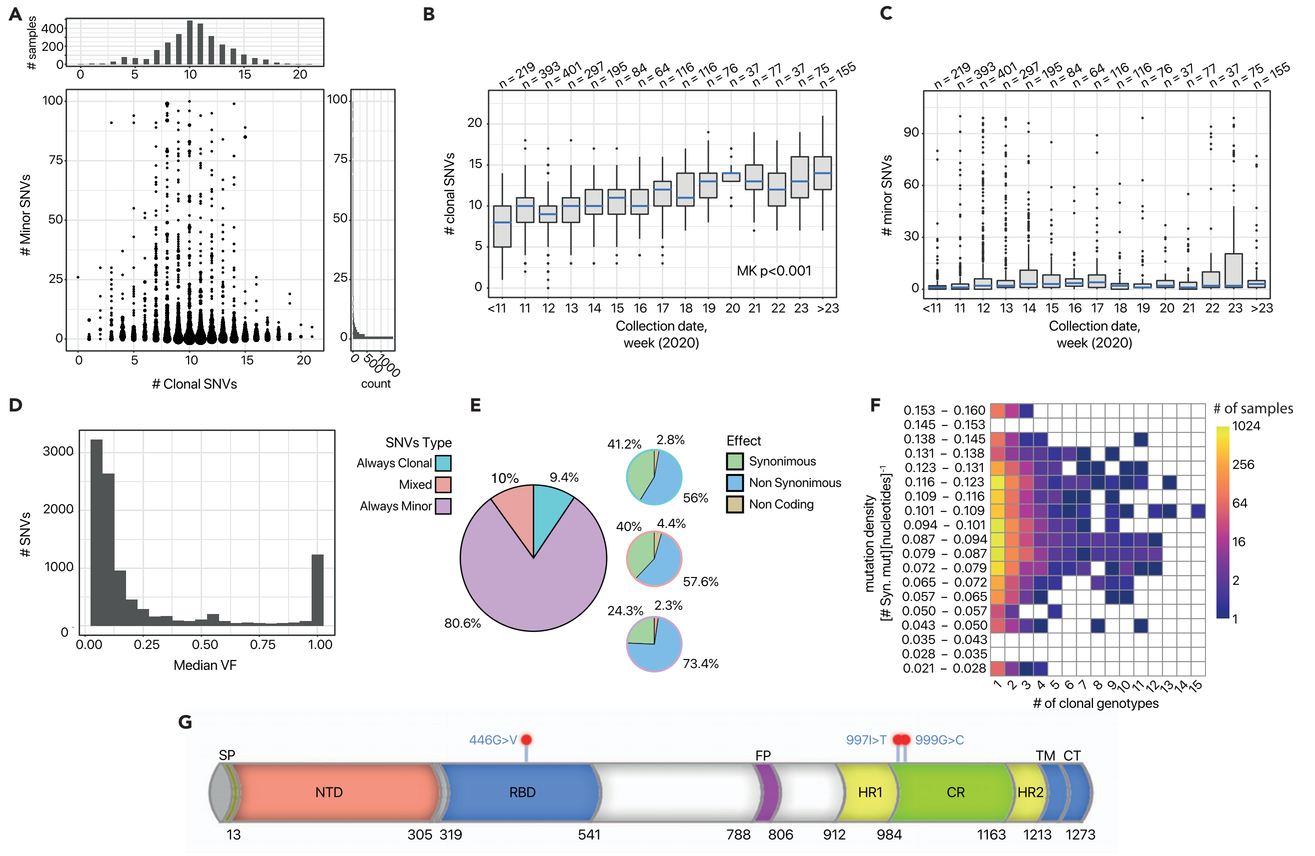

Figure 5. Mutational landscape of 2906SARS-CoV-2 samples (dataset #1)

(A) Scatterplot displaying, for each sample, the number of clonal (VF > 90%) and minor variants (VF ≤ 90%, node size proportional to the number of samples).

(B and C) Boxplots returning the distribution of the number of clonal (B) and minor variants (C), obtained by grouping samples according to collection date (weeks, 2020). The p value of the Mann-Kendall (MK) trend test on clonal variants is highly significant.

(D) Distribution of the median VF for all SNVs detected in the viral populations.

(E) Pie charts returning (left) the proportion of SNVs detected as always clonal, always minor, or mixed; (right) for each category, the proportion of synonymous, nonsynonymous, and non-coding variants (check the pie-chart border color for a visual clue).

(F) Heatmap returning the distribution of always minor SNVs with respect to (x axis) the number of clonal genotype of the phylogenomic model in Figure 3 in which each variant is observed, (y axis) the mutational density of the genome region in which it is located (see the Supplemental experimental procedures).

(G) Mapping of the candidate homoplastic minor variants located on the spike gene of the SARS-CoV-2 virus.

When the receptor binding domain (RBD) binds to ACE2 receptor on the target cell, it causes a conformational change responsible for the insertion of the fusion peptide (FP) into the target cell membrane. This, in turn, triggers further conformational changes, eventually promoting a direct interaction between HR1 trimer and HR2, which occurs upon bending of the flexible CR, in order to form a six-helical HR1-HR2 complex known as the fusion core region (FCR) in close proximity to the target cell plasma membrane, ultimately leading to viral fusion and cell entry.[92]

Peptides derived from the HR2 heptad region of enveloped viruses and able to efficiently bind to the viral HR1 region inhibit the formation of the FCR and completely suppress viral infection.[93] Therefore, the formation of the FCR is considered to be vital to mediate virus entry in the target cells, promoting viral infectivity. Of note, the CR is highly conserved across the Gammacoronavirus genus, supporting the notion that this region may play a very important but still unclear functional role (Figure 5G). Although structural and *in vitro* models will be required in order to extensively characterize the functional effect of these variants, the evidence that two of our three minor variants detected in the spike protein falls in a small domain comprising less than 14% of the entire spike protein length is intriguing, as it suggests a potential functional role for these mutations. It will be important to track the prevalence of these mutations, as well as of all other candidate convergent variants falling on different region of the SARS-CoV-2, to highlight possible transitions to clonality (see below). We also remark that, being a data-science computational approach, VERSO can struggle in dissecting complex mutational cases, since all the experimental hypotheses that can be generated are clearly data dependent. For this reason, and given the heterogeneity and limitations of currently available SARS-CoV-2 datasets, any hypothesis delivered by VERSO requires additional independent investigations and *ad hoc* experimental validations.

Mutational landscape

We analyzed in depth the mutational landscape of the samples of dataset #1. First, the comparison of the number of clonal

(VF > 90%) and minor variants detected in each host (Figure 5A) reveals a bimodal distribution of clonal variants (with first mode at 4 and s mode at 10), whereas minor variants display a more dispersed long-tailed distribution with median equal to 2 and average ≈23. From the plot, it is also clear that individuals characterized by the same clonal genotype may display a significantly different number of minor variants, with distinct distributions observed across clonal genotypes.

The comparison of the distribution of the number of variants obtained by grouping the samples with respect to collection week (Figures 5B and 5C) allows us to highlight a highly statistically significant increasing trend for clonal variants (Mann-Kendall trend test on median number of clonal variants, $p < 0.001$). This result would strongly support both the hypothesis of accumulation of clonal variants in the population and that of a concurrent increase of overall genomic diversity of SARS-CoV-2,[36,94] whereas the relevance of this phenomenon on minor variants is unclear.

We then focused on the properties of the SNVs detected in the population. Surprisingly, the distribution of the median VF for each detected variant (Figure 5D) reveals a bimodal distribution, with the large majority of variants showing either a very low or a very high VF, with only a small proportion of variants showing a median VF within the range 10%–90%. This behavior is typical of systems where the prevalence of some subpopulations is driven by positive Darwinian selection while others are purified.[95]

In order to analyze the two components of this distribution, we further categorized the variants as always clonal (i.e., SNVs detected with VF >90% in all samples), always minor (i.e., SNVs detected with VF 5% and ≤90% in all samples), and mixed (i.e., SNVs detected as clonal in at least one sample and as minor in at least another sample). As one can see in Figure 5E, 9.4%, 80.6%, and 10% all SNVs are respectively detected as always clonal, always minor, and mixed in our dataset. Moreover, 56%, 73.4%, and 57.6% of always clonal, always minor, and mixed variants, respectively, are nonsynonymous, whereas the large majority of remaining variants are synonymous.

These results would suggest that, in most cases, randomly emerging SARS-CoV-2 minor variants tend to remain at a low frequency in the population, whereas, in some circumstances, certain variants can undergo frequency increases and even become clonal, due to undetected mixed transmission events or to selection shifts, as it was observed by Poon et al.[8] for the cases of H3N2 and H1N1/2009 influenza. Interestingly, 15 variants identified as possibly convergent (see above) fall into this category and deserve further investigations (see Table S6 for additional details).

Transmission bottleneck analysis

The estimation of transmission bottlenecks might be of specific interest during the current pandemics. Despite most available methods requiring data collected on donor-host couples (see, e.g., Sobel Leonard et al.[96] and Ghafari et al.[97]), here we employed a strategy akin to Monsion et al.[98] and Lequime et al.[99] that is roughly based on the analysis of the variation of the VF variance of a number of candidate neutral mutations. The intuition is that variance shrinking indicates significant transmission bottlenecks, which, accordingly, would result in lower viral diversity transferred from a host to another and, possibly, in purification of certain variants in the population. As the analysis ideally requires the comparison of groups in which infection events have occurred, here we considered groups of samples with distinct clonal genotypes, separately. We then selected a number of variants as neutral markers. The rationale is that transmission phenomena such as bottlenecks are expected to significantly affect the VF variance of neutral markers (please see Supplemental experimental procedures for further details).

More in detail, we first split the samples of each clonal genotype for which a collection date is available into non-overlapping groups corresponding to two consecutive time windows; i.e., before and after the 14th week, 2020. Accordingly, three SNVs were selected as candidate-neutral or quasineutral markers, namely variants g.634T>C, g.14523A>G, and g.15168G>A. In Figure S6, one can find the distribution of the VF of the selected markers with respect to the time windows, which highlights moderate variations of the variance for all markers (see also Table S7. All in all, this result would suggest the presence of mild bottleneck effects, consistent with recent studies involving donor-host data.[43]

Application of VERSO to 2,766 samples from RNA-sequencing data (dataset #2)

We retrieved the raw Illumina RNA-sequencing data of 2,766 samples included in dataset #2 and applied VERSO to the mutational profiles of 1,438 samples selected after quality check. Twenty-three clonal variants were employed in the analysis, according to the filters described later.

The resulting phylogenetic model is consistent with the one obtained for dataset #1, despite minor differences (Figure S8A). Specifically, 18 distinct clonal genotypes are identified by VERSO step #1, 11 of which are identical to those found in the analysis of dataset #1 (in such cases the same genotype label was maintained; see Data S3 for the mapping with the lineage nomenclature proposed by Rambaut et al.[78]). Five further clonal genotypes are evolutionarily consistent and represent independent branches detected due to the non-overlapping composition of the dataset, and are labeled with progressive letters from the closest genotype (i.e., G13a, G21a, G22a, G22b, G23a), while the two samples of genotype G13a* might be safely assigned to genotype G13a, since the absence of mutation g.3037C>T is likely due to low coverage.

By excluding the remaining clonal genotype GH, which presents inconsistencies due to the presence of the candidate homoplastic variant g.11083G>T (*ORF1ab*, p.3606L>F, see above), all clonal genotypes display the same ordering in both datasets (also see the expanded clonal variant tree in Figure S9). This proves the robustness of the results delivered by VERSO step #1 even when dealing with data generated from distinct sequencing platforms.

By looking at the geo-temporal localization of samples obtained via Microreact[81] (Figure S8B), one can see that that dataset #2 includes samples with a significantly different geographical distribution with respect to dataset #1. This dataset contains sample from 10 countries, with the large majority collected in the United States (96.8%). More in detail, the samples of such countries are mostly characterized by clonal genotype G21. We further notice that, also for dataset #2, mutation g.23403A>G (*S*,p.614D>G) becomes prevalent in the population at late collection dates. Moreover, only samples belonging to previously defined type B are detected in this dataset.

The analysis of the intra-host genomic diversity was also performed for dataset #2 via VERSO step #2, which would suggest the existence of undetected infection events and of several infection clusters with distinct properties, even though no contact tracing is available in this case. Overall, this proves the general applicability of the VERSO framework, which can produce meaningful results when applied to data produced with any sequencing platforms. However, in order to minimize the possible impact of data- and platform-specific biases, our suggestion is to perform the VERSO analysis on datasets generated from different protocols separately.

Scalability

We finally assessed the computational time required by VERSO in a variety of simulated scenarios. The results are shown in the Supplemental experimental procedures (Figure S10) and demonstrate the scalability of VERSO also when processing large-scale datasets.

DISCUSSION

We introduced VERSO, a comprehensive framework for the high-resolution characterization of viral evolution from sequencing data, which is an improvement on currently available methods for the analysis of consensus sequences. VERSO exploits the distinct properties of clonal and minor variants to dissect the complex interplay of genomic evolution within hosts and transmission among hosts.

On the one hand, the probabilistic framework underlying VERSO step #1 delivers highly accurate and robust phylogenetic models from clonal variants, also in conditions of noisy observations and sampling limitations, as proved by extensive simulations and by the application to two large-scale SARS-CoV-2 datasets generated from distinct sequencing platforms. On the other hand, the characterization of intra-host genomic diversity provided by VERSO step #2 allows one to identify undetected infection paths, which were in our case validated with contact tracing data, as well as to intercept variants involved in homoplasies.

This may represent a major advancement in the analysis of viral evolution and spread and should be quickly implemented in combination with data-driven epidemiological models to deliver a high-precision platform for pathogen detection and surveillance.[12,100] This might be particularly relevant for countries that suffered outbreaks of exceptional proportions and for which the limitations and inhomogeneity of diagnostic tests have proved insufficient to define reliable descriptive/predictive models of disease diffusion. For instance, it was hypothesized that the rapid diffusion of COVID-19 might be likely due to the extremely high number of untested asymptomatic hosts.[101]

More accurate and robust phylogenetic models may allow one to improve the assessment of molecular clocks and, accordingly, the estimation of the parameters of epidemiological models such as susceptible-infected-recovered (SIR) and susceptible-infected-susceptible (SIS),[11,102] as well as to unravel the cryptic transmission paths.[8,12,13,103] Furthermore, the finer grain of the analysis on intra-host genomic similarity from sequencing data might be employed to enhance the active surveillance; for instance, by facilitating the identification of infection clusters and super-spreaders.[104] Finally, the characterization of variants possibly involved in positive selection processes might be used to drive the experimental research on treatments and vaccines.

EXPERIMENTAL PROCEDURES

Resource availability

Lead contact

Alex Graudenzi, Institute of Molecular Bioimaging and Physiology, Consiglio Nazionale delle Ricerche (IBFM-CNR), via F.lli Cervi, 93, 20,090 Segrate, Milan, Italy. alex.graudenzi@ibfm.cnr.it.

Materials availability

This study did not generate new unique reagents.

Data and code availability

VERSO is freely available at this link: https://github.com/BIMIB-DISCo/VERSO. VERSO step #1 is provided as an open source standalone R tool, whereas step #2 is provided as a Python script. The source code to replicate all the analyses presented in the manuscript, both on simulated and real-world datasets, is available at this link: https://github.com/BIMIB-DISCo/VERSO-UTILITIES.

SCANPY[59] is available at this link: https://scanpy.readthedocs.io/en/stable/. The Web-based tool for the geo-temporal visualization of samples, Microreact,[81] is available at this link: https://microreact.org/showcase. The tool employed to plot the phylogenomic model returned by VERSO step #1 (in Newick file format) is FigTree[80] and is available at this link: http://tree.bio.ed.ac.uk/software/figtree/. The tool used for the mapping between clonal genotype labels and the dynamic nomenclature proposed by Rambaut et al.[78] is pangolin 2.0[79] and is available at this link: https://github.com/cov-lineages/pangolin.

VERSO step #1: robust phylogenomic inference from clonal variant profiles

VERSO is a novel framework for the reconstruction of viral evolution models from raw sequencing data of viral genomes. It includes a two-step procedure, which we describe in the following.

The first step of VERSO employs a probabilistic maximum-likelihood framework for the reconstruction of robust phylogenetic trees from binarized mutational profiles of clonal variants (or, alternatively, from consensus sequences). This step relies on an evolved version of the algorithmic framework introduced by Ramazzotti et al.[105] for the inference of cancer evolution models from single-cell sequencing data, and can be executed independently from step #2, in case raw sequencing data are not available.

Inputs

The method takes as input a n (samples) $\times m$ (variants) binary mutational profile matrix, as defined on the basis of clonal SNVs only. In this case, an entry in a given sample is equal to 1 (present) if the VF is larger than a certain threshold (in our analyses, equal to 90%), it is equal to 0 if lower than a distinct threshold (in our analyses, equal to 5%), and is considered as missing (NA) in the other cases, thus modeling possible uncertainty in sequencing data or low coverage.

Notice that consensus sequences can be processed by VERSO step #1 by generating a consistent binarized mutational profile matrix. We also note that, given the intrinsic challenges associated with a reliable identification of low VF indels, the analysis focuses only on SNVs. Further details on the variant calling pipeline employed in this study are provided next.

The algorithmic framework

VERSO step #1 is a probabilistic framework that solves a Boolean matrix factorization problem with perfect phylogeny constraints and relying on the infinite sites assumption (ISA).[106,107] The ISA subsumes a consistent process of accumulation of clonal variants characterizing the evolutionary history of the virus and does not allow for losses of mutations or convergent variants (i.e., mutations observed in distinct clades).

In this regard, we recall that that the variant accumulation hypothesis holds only when considering clonal mutations. In fact, clonal mutations (e.g., A, B, C, D, and E) are present, by definition, in the large majority of the quasispecies of a given sample, depending on the chosen VF threshold (in our case, equal to 90%; see above). Since such variants are rarely lost, they are most likely transmitted from one host to another during infections. In addition, the origination of

new clonal mutations in single samples leads to the definition of new clonal genotypes, following a standard branching process (e.g., A, AB, ABC, ABD, ABDE). As a result, clonal mutations typically accumulate during the evolutionary history of a virus, excluding complex scenarios involving reticulation events,[53] whereas clonal genotypes can clearly become extinct. Conversely, variants with lower frequency do not necessarily accumulate, due to the high recombination rates, as well as to bottlenecks, founder effects, and stochasticity,[31] and this is the reason why they were considered separately in the analysis, via VERSO step #2 (see below).

More in detail, VERSO step #1 accounts for uncertainty in the data, by employing a maximum-likelihood approach (via Markov chain Monte Carlo [MCMC] search) that allows for the presence of false-positives, false-negatives, and missing data points in clonal variant profiles. As shown by Ramazzotti et al.[105] in a different experimental context, our algorithmic framework ensures robustness and scalability also in case of high rates of errors and missing data, due, for instance, to sampling limitations. Furthermore, it is robust to mild violations of the ISA (e.g., due to reticulation events, such as convergent variants) or mutation losses, which can be characterized after the inference, if present (see the specific features on homoplasy detection discussed next). Please refer to the Supplemental experimental procedures for further details on the algorithmic framework and its assumptions, including the probabilistic graphical model depicted in Figure S1 and the summary of notation in Table S4.

Outputs

The inference returns a set of maximum-likelihood variants trees (minimum 1) as sampled during the MCMC search, representing the ordering of accumulation of clonal variants, and a set of maximum-likelihood attachments of samples to variants. Given the variants tree and the maximum-likelihood attachments of samples to variants, VERSO outputs (1) a phylogenetic model where each leaf correspond to a sample, whereas internal nodes correspond to accumulating clonal variants; (2) the corrected clonal genotype of each sample (i.e., the binary mutational profile on clonal variants obtained after removing false-positives, false-negatives, and missing data).

The model naturally includes polytomies, which group samples with the same corrected clonal genotype. The length of the branches in the model represents the number of clonal substitutions (which can be normalized with respect to genome length), as in standard phylogenomic models, and the clades of the model correspond to viral lineages. The VERSO phylogenetic model is provided as output in Newick file format and can be processed and visualized in standard tools for phylogenetic analysis, such as FigTree[80] or Dendroscope.[108] Furthermore, VERSO allows one to visualize the geo-temporal localization of clonal genotypes via Microreact.[81]

Additional feature: homoplasy detection on clonal variants

Violations of the ISA are possible and can be due to reticulation events[53] such as homoplasies (i.e., identical variants detected in samples belonging to different clades) or to rare occurrences involving mutation losses (e.g., due to recombination-related deletions or to multiple mutations hitting an already mutated genome location[34]), as well as to infrequent transmission phenomena, such as super-infections[65,66] (a discussion on the general limitations of approaches based on phylogenetic trees when dealing with reticulation events is available elsewhere[109–111]).

In this regard, VERSO allows one to identify clonal mutations likely involved in homoplasies, in a similar fashion to the plethora of works on mitochondrial evolution and phylogenetic networks (discussed elsewhere[53,54,56,112–114]). In detail, given the maximum-likelihood phylogenetic tree, VERSO can estimate the variants that are theoretically expected in each sample. By comparing the theoretical observations with the input data, VERSO can estimate the rate of false-positives (i.e., the variants that are observed in the data but are not predicted by VERSO), and false-negatives (i.e., variants that are not observed but predicted). Variants that show particularly high estimated error rates represent candidate homoplasies and are flagged. First, this allows one to pinpoint samples exhibiting homoplastic mutations (see Figures 3 and S8) and, second, to reconstruct an expanded clonal variants tree, in which candidate homoplastic mutations are duplicated after the inference, so to allow the visualization of reticulation events, as proposed by Skála and Zrzavỳ[112] (see, e.g., Figures S3 and S9).

Furthermore, once this procedure has been completed, the list of flagged variants can include (1) mutations falling in highly mutated regions due to mutational hotspots, (2) phantom mutations (i.e., systematic artifacts generated during sequencing processes[56]), or (3) mutations that have been positively selected in the population (e.g., due to a particular functional advantage).

Since one might be interested in identifying positively selected mutations, VERSO allows one to perform a consecutive analysis that aims at highlighting the mutation-prone regions of the genome and that might be due to mutational hotspots or phantom mutations (see the Supplemental experimental procedures for further details). We finally note that the detection of homoplasies for minor variants requires a different algorithmic procedure, which is detailed in the following.

VERSO step #2: characterization of intra-host genomic diversity

In the second step, VERSO takes into account the VF profiles of groups of samples with the same corrected clonal genotype (identified via VERSO step #1), in order to characterize their intra-host genomic diversity and visualize it on a low-dimensional space. This allows one to highlight patterns of co-occurrence of minor variants, possibly underlying undetected infection events, as well as homoplasies involving; e.g., positively selected variants. Notice that this step requires raw sequencing data and the prior execution of step #1.

Inputs

VERSO step #2 takes as input a n (samples)$\times m$(variants) VF profile matrix, in which each entry includes the VF $\in (0,1)$ of a given mutation in a certain sample, after filtering out (1) the clonal variants employed in step #1 and (2) the minor variants possibly involved in homoplasies (see below). The variant calling pipeline employed in this work is detailed next.

The algorithmic framework

While it is sound to binarize clonal variant profiles to reconstruct a phylogenetic tree, it is opportune to consider the VF profiles when analyzing intra-host variants, for several reasons. First, VF profiles describe the intra-host genomic diversity of any given host, and this information would be lost during binarization. Second, minor variant profiles might be noisy, due to the relatively low abundance and to the technical limitations of sequencing experiments. Accordingly, such data may possibly include artifacts, which can be partially mitigated during the quality-check phase and by including in the analysis only highly confident variants. However, binarization with arbitrary thresholds might increase the false-positive rate, compromising the accuracy of any downstream analysis. Third, as specified above, the extent of transmission of minor variants among individuals is still partially obscure. The VF of minor variants is, in fact, highly affected by recombination processes, as well as by complex transmission phenomena, involving stochastic fluctuations, bottlenecks, and founder effects, which may lead certain variants changing their VF, not being transmitted, or even becoming clonal in the infected host.[57] The latter issue also suggests that the hypothesis of accumulation of minor variants during infections may not hold and should be relaxed.

For these reasons, VERSO step #2 defines a pairwise genomic distance, computed on the VF profiles, to be used in downstream analyses. The intuition is that samples displaying similar patterns of co-occurrence of minor variants might have a similar quasispecies architecture, thus being at a small evolutionary distance. Accordingly, this might indicate a direct or indirect infection event. In particular, in this work we employed the Bray-Curtis dissimilarity, which is defined as follows: given the ordered VF vectors of two samples (i.e. $v_i = \{VF_1^i, \ldots, VF_r^i,\}$ and $v_j = \{VF_1^j, \ldots, VF_r^j,\}$), the pairwise Bray-Curtis dissimilarity $d(i,j)$ is given by:

$$d(v_i, v_j) = \frac{\sum_{l=1}^{r} \left| VF_l^i - VF_l^j \right|}{\sum_{l=1}^{r} \left| VF_l^i + VF_l^j \right|}. \quad \text{(Equation 1)}$$

Since this measure weights the pairwise VF dissimilarity on each variant with respect to the sum of the VF of all variants detected in both samples, it can be effectively used to compare the intra-host genomic diversity of samples, as proposed, for instance, by Srinivas et al.[115] However, VERSO allows one to employ different distance metrics on VF profiles, such as correlation or Euclidean distance.

As a design choice, in VERSO, the genomic distance is computed among all samples associated to any given corrected clonal genotype, as inferred in step #1. The rationale is that, in a statistical inference framework modeling a complex interplay involving heterogeneous dynamical processes, it is crucial to stratify samples into homogeneous groups, to reduce the impact of possible

confounding effects.[116] Furthermore, as specified above, due to the distinct properties of clonal and minor variants during transmission, it is reasonable to assume that the event in which certain minor variants and no clonal variants are transmitted from a host to another during the infection is extremely unlikely. Accordingly, the clonal variants employed for the reconstruction of the phylogenetic tree in step #1 are excluded from the computation of the intra-host genomic distance among samples.

In order to produce useful knowledge from the genomic distance discussed above and since, in real-world scenarios, this is a typically complex high-dimensional problem, it is sound to employ state-of-the-art strategies for dimensionality reduction and (sample) clustering, as typically done in single-cell analyses.[117] In this regard, the workflow employed in VERSO ensures high scalability with large datasets, also making it possible to take advantage of effective analysis and visualization features. In detail, the workflow includes three steps: (1) the computation of k-NNG, which can be executed on the original VF matrix, or after applying PCA, to possibly reduce the effect of noisy observations (when the number of samples and variants is sufficiently high); (2) the clustering of samples via either Louvain or Leiden algorithms for community detection;[118] (3) the projection of samples on a low-dimensional space via standard tSNE[68] or UMAP[67] plots.

Outputs

As output, VERSO step #2 delivers both the partitioning of samples in homogeneous clusters and the visualization in a low-dimensional space, also allowing samples to be labeled according to other covariates, such as collection date or geographical location. In the map in Figure 3, for instance, the intra-host genomic diversity of each sample and the genomic distance among samples are projected on the first two UMAP components, whereas samples that are connected by k-NNG edges display similar patterns of co-occurrence of variants. Accordingly, the map shows clusters of samples likely affected by infection events, in which (a fraction of) quasispecies might have been transmitted from one host to another. This represents a major novelty introduced by VERSO and also allows one to effectively visualize the space of VF profiles.

To facilitate the usage, VERSO step #2 is provided as a Python script which employs the SCANPY suite of tools,[59] which is typically used in single-cell analyses and includes a number of highly effective analysis and visualization features.

Additional feature: homoplasy detection on minor variants

Also in the case of minor variants, it is important to pinpoint possible homoplasies that might be due to mutational hotspots, phantom mutations, and convergent variants. Given the phylogenetic model retrieved via step #1, VERSO allows one to flag the variants that are detected in a number of clonal genotypes exceeding a user-defined threshold. In our case, the threshold is equal to 1, meaning that all minor variants found in more than one clonal genotype are flagged.

Such variants are then excluded from the computation of the intra-host genomic distance, prior to the execution of step #2. Furthermore, the list of flagged variants can be investigated as proposed for step #1 (see above), in order to possibly identify mutations involved in positive selection scenarios.

Datasets description

Dataset #1 (Illumina Amplicon sequencing)

We analyzed 3,960 samples from distinct individuals obtained from 22 NCBI BioProjects, which, at the time of writing, are all the publicly available datasets including raw Illumina Amplicon sequencing data. In detail, we selected the following NCBI BioProjects: (1) PRJNA613958, (2) PRJNA614546, (3) PRJNA616147, (4) PRJNA622817, (5) PRJNA623683, (6) PRJNA625551, (7) PRJNA627229, (8) PRJNA627662, (9) PRJNA629891, (10) PRJNA631042, (11) PRJNA633948, (12) PRJNA634119, (13) PRJNA636446, (14) PRJNA636748, (15) PRJNA639066, (16) PRJNA643575, (17) PRJNA645906, (18) PRJNA647448, (19) PRJNA647529, (20) PRJNA650037, (21) PRJNA656534, and (22) PRJNA656695.

Dataset #2 (Illumina RNA sequencing)

We analyzed 2,766 samples from distinct individuals obtained from 22 NCBI BioProjects, which, at the time of writing, are all the publicly available datasets including raw Illumina RNA-sequencing data. In detail, we selected the following NCBI BioProjects: (1) PRJNA601736, (2) PRJNA603194, (3) PRJNA605983, (4) PRJNA607948, (5) PRJNA608651, (6) PRJNA610428, (7) PRJNA615319, (8) PRJNA616446, (9) PRJNA623895, (10) PRJNA624792, (11) PRJNA626526, (12) PRJNA631061, (13) PRJNA636446, (14) PRJNA637892, (15) PRJNA639591, (16) PRJNA639864, (17) PRJNA650134, (18) PRJNA650245, (19) PRJNA655577, (20) PRJNA657938, (21) PRJNA657985, and (22) PRJNA658211.

Contact tracing data

Contact tracing data were obtained from the study presented by Rockett et al.[69] In detail, for 65 samples included in dataset #1 (NCBI BioProject: PRJNA633948), information on households, work institutions, and epidemiological linkages are provided. Thus, it is possible to identify three different contact groups based on institutions regularly frequented by patients and one-household couples. Contact information was employed to assess the relation between the intra-host genomic similarity and the contact dynamics. The results are provided in the main text.

Parameter settings

Parameter settings of variant calling (datasets #1 and #2)

We converted all the samples to FASTQ files using the Sequence Read Archive (SRA) toolkit. Following Bastola et al.,[75] we used Trimmomatic (version 0.39) to remove the nucleotides with low quality score from the RNA sequences with the following settings: LEADING:20 TRAILING:20 SLIDINGWINDOW:4:20 MINLEN:40. We then used bwa mem (version 0.7.17) to map reads to SARS-CoV-2-ANC reference genome (Data S1; see Results). We generated sorted BAM files from bwa mem results using SAMtools (version 1.10) and removed duplicates with Picard (version 2.22.2). Variant calling was performed generating mpileup files using SAMtools and then running VarScan (min-var-freq parameter set to 0.01).[119]

We note that it was recently reported that some currently available SARS-CoV-2 datasets exhibit quality issues.[13,120] Accordingly, one should be extremely careful when performing quality check and, especially, when considering low-frequency variants, which might possibly result from sequencing artifacts even in case of high-coverage experiments. In this regard, many effective approaches can be employed to reduce false variants. For instance, the Broad Institute recently updated an effective variant calling pipeline for viral genome data,[121] while new methods for error correction of viral sequencing have been proposed at a widely used website (https://virological.org), which also includes a number of useful up-to-date guidelines and best practices for viral evolution analyses.

In our case, we here employed the following significance filters on variants. In particular, we kept only the mutations (1) showing a VarScan significance p value <0.01 (Fisher's exact test on the read counts supporting reference and variant alleles) and more than 25 reads of support in at least 75% of the samples, (2) displaying a VF >5%. As a result, we selected a list of 15,892 (over 55,280 overall SNVs) highly confident SNVs for dataset #1 and 7,389 (over 53,354) for dataset #2.

High-quality variants were then mapped on SARS-CoV-2 coding sequences (CDSs) via a custom R script, also by highlighting synonymous/nonsynonymous states and amino acid substitutions for the related open reading frame (ORF) product. In particular, we translated reference and mutated CDSs with the seqinr R package to obtain the relative amino acid sequences, which we compared to assess the effect of each nucleotide variation in terms of amino acid substitution.

We finally note that availability of the cycle threshold (Ct) values generated by qPCR and the related quantification of the amounts of viral transcripts would be very useful to characterize samples with high viral load, yet this information is not available for the considered datasets.

Quality check (datasets #1 and #2)

In order to select high-quality samples, we selected only those exhibiting high coverage and in particular those with at least 25 reads in more than 90% of the SARS-CoV-2-ANC genome. In addition, we filtered out all samples exhibiting more than 100 minor variants (VF $\leq$ 90%).

We finally excluded samples SRR11597146 and SRR11476447 from dataset #1, as the first sample displays zero SNVs and the second one reports an unfeasible collection date (i.e., 30th Jan. 2019).

After the quality-check filters, 2,906 samples of dataset #1 are left for downstream analyses, in which 10,571 distinct high-quality SNVs are observed, and 1,438 samples are left for dataset #2, with 6,143 high-quality SNVs.

Parameter settings of VERSO *(datasets #1 and #2)*

The phylogenomic analysis via VERSO step #1 was performed on datasets #1 and #2 by considering only clonal variants (VF > 90%) detected in at least 3% of the samples. A grid search comprising 16 different error rates was employed (see Table S3). Samples with the same corrected clonal genotype were grouped in polytomies in the final phylogenetic models.

The analysis of the intra-host genomic diversity via VERSO step #2 was performed by considering the VF profiles of all samples, by excluding (1) the clonal variants employed in the phylogenomic reconstruction via VERSO step #1, (2) the minor variants involved in homoplasies (i.e., observed in more than one clonal genotype returned by VERSO step #1). Missing values (NA) were imputed to 0 for downstream analysis. A number of principal components equals to 10 was employed in PCA step, prior to the computation of the k-NNG (k = 10) on the Bray-Curtis dissimilarity of VF profiles. Leiden algorithm was applied with resolution = 1 (see Table S3 for the parameter settings of VERSO employed in the case studies).

Parameter settings of simulations

In order to compare the performance of VERSO step #1 with competing phylogenomic tools (i.e., IQ-TREE[10] and BEAST 2[22]), we performed extensive simulations via msprime,[70] which simulates a backwards-in-time coalescent model.

In particular, we simulated 20 distinct evolutionary processes, with the following parameters: n = 1,000 total samples, effective population size N_e = 0.5 (i.e., haploid population), mutational rate $M = 2 \times 10^{-6}$ mutations per site per generation, and a genome of length L = 29,903 bases. Such parameters were chosen to roughly approximate the mutational rate currently estimated for SARS-CoV-2 (i.e., $M \approx 10^{-3}$ mutations per site per year and $\approx 10^{-3}\frac{\text{generation}}{\text{year}}$[122]) and to obtain a number of clonal mutations (in the range 15–30) that is comparable with the one observed in the real-word scenarios (see the case studies). As output, msprime returns a phylogenetic tree representing the genealogy between the samples, the genotype of all samples (i.e., the leaves of the tree), and the location of all mutations.

The genotypes of the samples were then inflated with different levels of noise, with false-positive rate α and false-negative rate β (see the parameter settings in Table S1), in order to assess the performance of the methods in conditions of noisy observations and possible sequencing issues. Finally, we subsampled all datasets to obtain two distinct samples sizes (500 and 1,000 samples), in order to test the robustness of methods in conditions of sampling limitations.

The parameters of the phylogenetic methods employed in the comparative assessment are reported in the Supplemental experimental procedures (Table S2).

SUPPLEMENTAL INFORMATION

Supplemental Information can be found online at https://doi.org/10.1016/j.patter.2021.100212.

ACKNOWLEDGMENTS

This work was partially supported by the Elixir Italian Chapter and the SysBio-Net project, a Ministero dell'Istruzione, dell'UniversitÃ e della Ricerca initiative for the Italian Roadmap of European Strategy Forum on Research Infrastructures, and by AIRC-IG grant 22082. Partial support was also provided by the CRUK/AECC/AIRC Accelerator Award #22790, Single-cell Cancer Evolution in the Clinic. We thank Giulio Caravagna, Chiara Damiani, Lucrezia Patruno, and Francesco Craighero for helpful discussions. We also thank David Posada for interesting suggestions on the preliminary version of the manuscript.

AUTHOR CONTRIBUTIONS

D.R., F.A., D.M., A.G., and R.P. designed the approach. D.R., F.A., D.M., and A.G. defined, implemented, and executed the computational methods. D.R., F.A., D.M., and A.G. performed the simulations. D.R., F.A., D.M., C.G.-P., M.A., A.G., and R.P. analyzed the data and interpreted the results. R.P. supervised the experimental data analysis. A.G. and D.R. supervised the computational analysis. A.G. and R.P. drafted the manuscript, which all authors discussed, reviewed, and approved.

DECLARATION OF INTERESTS

The authors declare no competing interests.

Received: June 12, 2020
Revised: November 30, 2020
Accepted: January 22, 2021
Published: January 28, 2021

REFERENCES

1. Zhou, P., Yang, X.L., Wang, X.G., Hu, B., Zhang, L., Zhang, W., Si, H.R., Zhu, Y., Li, B., Huang, C.L., et al. (2020). A pneumonia outbreak associated with a new coronavirus of probable bat origin. Nature *579*, 270–273, https://doi.org/10.1038/s41586-020-2012-7.
2. Wu, F., Zhao, S., Yu, B., Chen, Y.M., Wang, W., Song, Z.G., Hu, Y., Tao, Z.W., Tian, J.H., Pei, Y.Y., et al. (2020). A new coronavirus associated with human respiratory disease in China. Nature *579*, 265–269, https://doi.org/10.1038/s41586-020-2008-3.
3. Andersen, K.G., Rambaut, A., Lipkin, W.I., Holmes, E.C., and Garry, R.F. (2020). The proximal origin of SARS-CoV-2. Nat. Med. *26*, 450–452, https://doi.org/10.1038/s41591-020-0820-9.
4. Xiao, K., Zhai, J., Feng, Y., Zhou, N., Zhang, X., Zou, J.J., Li, N., Guo, Y., Li, X., Shen, X., et al. (2020). Isolation of SARS-CoV-2-related coronavirus from Malayan pangolins. Nature *583*, 286–289, https://doi.org/10.1038/s41586-020-2313-x.
5. Deng, X., Gu, W., Federman, S., du Plessis, L., Pybus, O.G., Faria, N.R., Wang, C., Yu, G., Bushnell, B., Pan, C.Y., et al. (2020). Genomic surveillance reveals multiple introductions of SARS-CoV-2 into Northern California. Science *369*, 582–587, https://doi.org/10.1126/science.abb9263.
6. Grubaugh, N.D., Petrone, M.E., and Holmes, E.C. (2020). We shouldn't worry when a virus mutates during disease outbreaks. Nat. Microbiol. *5*, 529–530, https://doi.org/10.1038/s41564-020-0690-4.
7. Grenfell, B.T., Pybus, O.G., Gog, J.R., Wood, J.L., Daly, J.M., Mumford, J.A., and Holmes, E.C. (2004). Unifying the epidemiological and evolutionary dynamics of pathogens. Science *303*, 327–332, https://doi.org/10.1126/science.1090727.
8. Poon, L.L., Song, T., Rosenfeld, R., Lin, X., Rogers, M.B., Zhou, B., Sebra, R., Halpin, R.A., Guan, Y., Twaddle, A., et al. (2016). Quantifying influenza virus diversity and transmission in humans. Nat. Genet. *48*, 195, https://doi.org/10.1038/ng.3479.
9. Shu, Y., and McCauley, J. (2017). GISAID: global initiative on sharing all influenza data–from vision to reality. Euro Surveill. *22*, https://doi.org/10.2807/1560-7917.ES.2017.22.13.30494.
10. Nguyen, L.T., Schmidt, H.A., Von Haeseler, A., and Minh, B.Q. (2015). IQ-TREE: a fast and effective stochastic algorithm for estimating maximum-likelihood phylogenies. Mol. Biol. Evol. *32*, 268–274, https://doi.org/10.1093/molbev/msu300.
11. Volz, E.M., Koelle, K., and Bedford, T. (2013). Viral phylodynamics. PLoS Comput. Biol. *9*, https://doi.org/10.1371/journal.pcbi.1002947.
12. Faria, N.R., Quick, J., Claro, I., Theze, J., de Jesus, J.G., Giovanetti, M., Kraemer, M.U., Hill, S.C., Black, A., da Costa, A.C., et al. (2017). Establishment and cryptic transmission of Zika virus in Brazil and the Americas. Nature *546*, 406–410, https://doi.org/10.1038/nature22401.
13. Worobey, M., Pekar, J., Larsen, B.B., Nelson, M.I., Hill, V., Joy, J.B., Rambaut, A., Suchard, M.A., Wertheim, J.O., and Lemey, P. (2020). The emergence of SARS-CoV-2 in Europe and North America. Science *370*, 564–570, https://doi.org/10.1126/science.abc8169.
14. Bandelt, H.J., Forster, P., and Röhl, A. (1999). Median-joining networks for inferring intraspecific phylogenies. Mol. Biol. Evol. *16*, 37–48, https://doi.org/10.1093/oxfordjournals.molbev.a026036.
15. O'Meara, B.C. (2012). Evolutionary inferences from phylogenies: a review of methods. Annu. Rev. Ecol. Evol. Syst. *43*, 267–285, https://doi.org/10.1146/annurev-ecolsys-110411-160331.

16. Ronquist, F., Teslenko, M., van der Mark, P., Ayres, D.L., Darling, A., Höhna, S., Larget, B., Liu, L., Suchard, M.A., and Huelsenbeck, J.P. (2012). MrBayes 3.2: efficient Bayesian phylogenetic inference and model choice across a large model space. Syst. Biol. *61*, 539–542, https://doi.org/10.1093/sysbio/sys029.

17. Stamatakis, A. (2014). RAxML version 8: a tool for phylogenetic analysis and post-analysis of large phylogenies. Bioinformatics *30*, 1312–1313, https://doi.org/10.1093/bioinformatics/btu033.

18. Didelot, X., Fraser, C., Gardy, J., and Colijn, C. (2017). Genomic infectious disease epidemiology in partially sampled and ongoing outbreaks. Mol. Biol. Evol. *34*, 997–1007, https://doi.org/10.1093/molbev/msw275.

19. Skums, P., Zelikovsky, A., Singh, R., Gussler, W., Dimitrova, Z., Knyazev, S., Mandric, I., Ramachandran, S., Campo, D., Jha, D., et al. (2018). Quentin: reconstruction of disease transmissions from viral quasispecies genomic data. Bioinformatics *34*, 163–170, https://doi.org/10.1093/bioinformatics/btx402.

20. De Maio, N., Worby, C.J., Wilson, D.J., and Stoesser, N. (2018). Bayesian reconstruction of transmission within outbreaks using genomic variants. PLoS Comput. Biol. *14*, e1006117, https://doi.org/10.1093/sysbio/sys029.

21. Kosakovsky Pond, S.L., Weaver, S., Leigh Brown, A.J., and Wertheim, J.O. (2018). HIV-TRACE (TRAnsmission Cluster Engine): a tool for large scale molecular epidemiology of HIV-1 and other rapidly evolving pathogens. Mol. Biol. Evol. *35*, 1812–1819, https://doi.org/10.1093/molbev/msy016.

22. Bouckaert, R., Vaughan, T.G., Barido-Sottani, J., Duchêne, S., Fourment, M., Gavryushkina, A., Heled, J., Jones, G., Kühnert, D., De Maio, N., et al. (2019). Beast 2.5: an advanced software platform for Bayesian evolutionary analysis. PLoS Comput. Biol. *15*, e1006650, https://doi.org/10.1371/journal.pcbi.1006650.

23. Lai, A., Bergna, A., Acciarri, C., Galli, M., and Zehender, G. (2020). Early phylogenetic estimate of the effective reproduction number of SARS-CoV-2. J. Med. Virol. *92*, 675–679, https://doi.org/10.1002/jmv.25723.

24. Forster, P., Forster, L., Renfrew, C., and Forster, M. (2020). Phylogenetic network analysis of SARS-CoV-2 genomes. Proc. Natl. Acad. Sci. U S A *117*, 9241–9243, https://doi.org/10.1073/pnas.2004999117.

25. Dong, R., Pei, S., Yin, C., He, R.L., and Yau, S.S.T. (2020). Analysis of the hosts and transmission paths of SARS-CoV-2 in the COVID-19 outbreak. Genes *11*, 637, https://doi.org/10.3390/genes11060637.

26. Nakhleh, L. (2009). A metric on the space of reduced phylogenetic networks. IEEE/ACM Trans. Comput. Biol. Bioinform. *7*, 218–222, https://doi.org/10.1126/10.1109/TCBB.2009.2.

27. Yuan, K., Sakoparnig, T., Markowetz, F., and Beerenwinkel, N. (2015). BitPhylogeny: a probabilistic framework for reconstructing intra-tumor phylogenies. Genome Biol. *16*, 36, https://doi.org/10.1186/s13059-015-0592-6.

28. Villabona-Arenas, C.J., Hanage, W.P., and Tully, D.C. (2020). Phylogenetic interpretation during outbreaks requires caution. Nat. Microbiol. *5*, 876–877, https://doi.org/10.1038/s41564-020-0738-5.

29. Mavian, C., Marini, S., Manes, C., Capua, I., Prosperi, M., and Salemi, M. (2020). Regaining perspective on SARS-CoV-2 molecular tracing and its implications. medRxiv. https://doi.org/10.1101/2020.03.16.20034470.

30. Domingo, E., Martínez-Salas, E., Sobrino, F., de la Torre, J.C., Portela, A., Ortín, J., López-Galindez, C., Pérez-Breña, P., Villanueva, N., Nájera, R., et al. (1985). The quasispecies (extremely heterogeneous) nature of viral RNA genome populations: biological relevance–a review. Gene *40*, 1–8, https://doi.org/10.1016/0378-1119(85)90017-4.

31. Domingo, E., Sheldon, J., and Perales, C. (2012). Viral quasispecies evolution. Microbiol. Mol. Biol. Rev. *76*, 159–216, https://doi.org/10.1128/MMBR.05023-11.

32. Acevedo, A., Brodsky, L., and Andino, R. (2014). Mutational and fitness landscapes of an RNA virus revealed through population sequencing. Nature *505*, 686–690, https://doi.org/10.1038/nature12861.

33. Novella, I.S., Domingo, E., and Holland, J.J. (1995). Rapid viral quasispecies evolution: implications for vaccine and drug strategies. Mol. Med. Today *1*, 248–253, https://doi.org/10.1016/s1357-4310(95)91551-6.

34. Simon-Loriere, E., and Holmes, E.C. (2011). Why do RNA viruses recombine? Nat. Rev. Microbiol. *9*, 617–626, https://doi.org/10.1038/nrmicro2614.

35. Graudenzi, A., Maspero, D., Angaroni, F., Piazza, R., and Ramazzotti, D. (2020). Mutational signatures and heterogeneous host response revealed via large-scale characterization of SARS-CoV-2 genomic diversity. iScience *24*, 102116.

36. Shen, Z., Xiao, Y., Kang, L., Ma, W., Shi, L., Zhang, L., Zhou, Z., Yang, J., Zhong, J., Yang, D., et al. (2020). Genomic diversity of SARS-CoV-2 in coronavirus disease 2019 patients. Clin. Infect. Dis. *71*, https://doi.org/10.1093/cid/ciaa203.

37. Wölfel, R., Corman, V.M., Guggemos, W., Seilmaier, M., Zange, S., Müller, M.A., Niemeyer, D., Jones, T.C., Vollmar, P., Rothe, C., et al. (2020). Virological assessment of hospitalized patients with COVID-2019. Nature *581*, 465–469, https://doi.org/10.1038/s41586-020-2196-x.

38. Capobianchi, M.R., Rueca, M., Messina, F., Giombini, E., Carletti, F., Colavita, F., Castilletti, C., Lalle, E., Bordi, L., Vairo, F., et al. (2020). Molecular characterization of SARS-CoV-2 from the first case of COVID-19 in Italy. Clin. Microbiol. Infect. *26*, 954–956, https://doi.org/10.1016/j.cmi.2020.03.025.

39. Rose, R., Nolan, D.J., Moot, S., Feehan, A., Cross, S., Garcia-Diaz, J., and Lamers, S.L. (2020). Intra-host site-specific polymorphisms of SARS-CoV-2 is consistent across multiple samples and methodologies. medRxiv. https://doi.org/10.1101/2020.04.24.20078691.

40. Lu, J., du Plessis, L., Liu, Z., Hill, V., Kang, M., Lin, H., Sun, J., François, S., Kraemer, M.U., Faria, N.R., et al. (2020). Genomic epidemiology of SARS-CoV-2 in Guangdong province, China. Cell *181*, 997–1003.e9, https://doi.org/10.1016/j.cell.2020.04.023.

41. Lythgoe, K.A., Hall, M.D., Ferretti, L., de Cesare, M., MacIntyre-Cockett, G., Trebes, A., Andersson, M., Otecko, N., Wise, E.L., Moore, N., et al. (2020). Shared SARS-CoV-2 diversity suggests localised transmission of minority variants. bioRxiv. https://doi.org/10.1101/2020.05.28.118992.

42. Seemann, T., Lane, C.R., Sherry, N.L., Duchene, S., Gonçalves da Silva, A., Caly, L., Sait, M., Ballard, S.A., Horan, K., Schultz, M.B., et al. (2020). Tracking the COVID-19 pandemic in Australia using genomics. Nat. Commun. *11*, 4376, https://doi.org/10.1038/s41467-020-18314-x.

43. Popa, A., Genger, J.W., Nicholson, M.D., Penz, T., Schmid, D., Aberle, S.W., Agerer, B., Lercher, A., Endler, L., Colaço, H., et al. (2020). Genomic epidemiology of superspreading events in Austria reveals mutational dynamics and transmission properties of SARS-CoV-2. Sci. Transl. Med. *12*, https://doi.org/10.1126/scitranslmed.abe2555.

44. Miralles, R., Gerrish, P.J., Moya, A., and Elena, S.F. (1999). Clonal interference and the evolution of RNA viruses. Science *285*, 1745–1747, https://doi.org/10.1126/science.285.5434.1745.

45. Xu, D., Zhang, Z., and Wang, F.S. (2004). SARS-associated coronavirus quasispecies in individual patients. N. Engl. J. Med. *350*, 1366–1367, https://doi.org/10.1056/NEJMc032421.

46. Wright, C.F., Morelli, M.J., Thébaud, G., Knowles, N.J., Herzyk, P., Paton, D.J., Haydon, D.T., and King, D.P. (2011). Beyond the consensus: dissecting within-host viral population diversity of foot-and-mouth disease virus by using next-generation genome sequencing. J. Virol. *85*, 2266–2275, https://doi.org/10.1128/JVI.01396-10.

47. Park, D., Huh, H.J., Kim, Y.J., Son, D.S., Jeon, H.J., Im, E.H., Kim, J.W., Lee, N.Y., Kang, E.S., Kang, C.I., et al. (2016). Analysis of intrapatient heterogeneity uncovers the microevolution of middle east respiratory syndrome coronavirus. Mol. Case Stud. *2*, a001214, https://doi.org/10.1101/mcs.a001214.

48. Ni, M., Chen, C., Qian, J., Xiao, H.X., Shi, W.F., Luo, Y., Wang, H.Y., Li, Z., Wu, J., Xu, P.S., et al. (2016). Intra-host dynamics of Ebola virus during

2014. Nat. Microbiol. *1*, 16151, https://doi.org/10.1038/nmicrobiol.2016.151.

49. Ramazzotti, D., Caravagna, G., Olde Loohuis, L., Graudenzi, A., Korsunsky, I., Mauri, G., Antoniotti, M., and Mishra, B. (2015). CAPRI: efficient inference of cancer progression models from cross-sectional data. Bioinformatics *31*, 3016–3026, https://doi.org/10.1093/bioinformatics/btv296.

50. Beerenwinkel, N., Schwarz, R.F., Gerstung, M., and Markowetz, F. (2014). Cancer evolution: mathematical models and computational inference. Syst. Biol. *64*, e1–e25, https://doi.org/10.1093/sysbio/syu081.

51. Caravagna, G., Graudenzi, A., Ramazzotti, D., Sanz-Pamplona, R., De Sano, L., Mauri, G., Moreno, V., Antoniotti, M., and Mishra, B. (2016). Algorithmic methods to infer the evolutionary trajectories in cancer progression. Proc. Natl. Acad. Sci. U S A *113*, E4025–E4034, https://doi.org/10.1073/pnas.1520213113.

52. Schwartz, R., and Schäffer, A.A. (2017). The evolution of tumour phylogenetics: principles and practice. Nat. Rev. Genet. *18*, 213–229, https://doi.org/10.1038/nrg.2016.170.

53. Posada, D., and Crandall, K.A. (2001). Intraspecific gene genealogies: trees grafting into networks. Trends Ecol. Evol. *16*, 37–45, https://doi.org/10.1016/S0169-5347(00)02026-7.

54. Boc, A., Diallo, A.B., and Makarenkov, V. (2012). T-rex: a web server for inferring, validating and visualizing phylogenetic trees and networks. Nucleic Acids Res. *40* (W1), W573–W579, https://doi.org/10.1093/nar/gks485.

55. Bull, J., Badgett, M., Wichman, H.A., Huelsenbeck, J.P., Hillis, D.M., Gulati, A., Ho, C., and Molineux, I. (1997). Exceptional convergent evolution in a virus. Genetics *147*, 1497–1507. https://www.genetics.org/content/147/4/1497.

56. Bandelt, H.J., Quintana-Murci, L., Salas, A., and Macaulay, V. (2002). The fingerprint of phantom mutations in mitochondrial DNA data. Am. J. Hum. Genet. *71*, 1150–1160, https://doi.org/10.1086/344397.

57. Gutierrez, S., Yvon, M., Pirolles, E., Garzo, E., Fereres, A., Michalakis, Y., and Blanc, S. (2012). Circulating virus load determines the size of bottlenecks in viral populations progressing within a host. PLoS Pathog. *8*, 1–10, https://doi.org/10.1371/journal.ppat.1003009.

58. Firestone, S.M., Hayama, Y., Bradhurst, R., Yamamoto, T., Tsutsui, T., and Stevenson, M.A. (2019). Reconstructing foot-and-mouth disease outbreaks: a methods comparison of transmission network models. Sci. Rep. *9*, 4809, https://doi.org/10.1038/s41598-019-41103-6.

59. Wolf, F.A., Angerer, P., and Theis, F.J. (2018). SCANPY: large-scale single-cell gene expression data analysis. Genome Biol. *19*, 15, https://doi.org/10.1186/s13059-017-1382-0.

60. Prosperi, M.C., and Salemi, M. (2012). Qure: software for viral quasispecies reconstruction from next-generation sequencing data. Bioinformatics *28*, 132–133, https://doi.org/10.1093/bioinformatics/btr627.

61. Giallonardo, F.D., Töpfer, A., Rey, M., Prabhakaran, S., Duport, Y., Leemann, C., Schmutz, S., Campbell, N.K., Joos, B., Lecca, M.R., et al. (2014). Full-length haplotype reconstruction to infer the structure of heterogeneous virus populations. Nucleic Acids Res. *42*, e115, https://doi.org/10.1093/nar/gku537.

62. Töpfer, A., Marschall, T., Bull, R.A., Luciani, F., Schönhuth, A., and Beerenwinkel, N. (2014). Viral quasispecies assembly via maximal clique enumeration. PLoS Comput. Biol. *10*, 1–10, https://doi.org/10.1371/journal.pcbi.1003515.

63. Barik, S., Das, S., and Vikalo, H. (2018). QSdpR: viral quasispecies reconstruction via correlation clustering. Genomics *110*, 375–381, https://doi.org/10.1016/j.ygeno.2017.12.007.

64. Knyazev, S., Hughes, L., Skums, P., and Zelikovsky, A. (2020). Epidemiological data analysis of viral quasispecies in the next-generation sequencing era. Brief. Bioinformatics, bbaa101, https://doi.org/10.1093/bib/bbaa101.

65. Alizon, S. (2013). Co-infection and super-infection models in evolutionary epidemiology. Interface Focus *3*, 20130031, https://doi.org/10.1098/rsfs.2013.0031.

66. Garcia-Vidal, C., Sanjuan, G., Moreno-García, E., Puerta-Alcalde, P., Garcia-Pouton, N., Chumbita, M., Fernandez-Pittol, M., Pitart, C., Inciarte, A., Bodro, M., et al. (2020). Incidence of co-infections and superinfections in hospitalized patients with COVID-19: a retrospective cohort study. Clin. Microbiol. Infect. *27*, 83–88, https://doi.org/10.1016/j.cmi.2020.07.041.

67. McInnes, L., Healy, J., Saul, N., and Großberger, L. (2018). UMAP: uniform manifold approximation and projection. J. Open Source Softw. *3*, 861, https://doi.org/10.21105/joss.00861.

68. van der Maaten, L., and Hinton, G. (2008). Visualizing data using t-SNE. J. Machine Learn. Res. *9*, 2579–2605. http://jmlr.org/papers/v9/vandermaaten08a.html.

69. Rockett, R.J., Arnott, A., Lam, C., Sadsad, R., Timms, V., Gray, K.A., Eden, J.S., Chang, S., Gall, M., Draper, J., et al. (2020). Revealing COVID-19 transmission in Australia by SARS-CoV-2 genome sequencing and agent-based modeling. Nat. Med. *26*, 1398–1404, https://doi.org/10.1038/s41591-020-1000-7.

70. Kelleher, J., Etheridge, A.M., and McVean, G. (2016). Efficient coalescent simulation and genealogical analysis for large sample sizes. PLoS Comput. Biol. *12*, 1–22, https://doi.org/10.1371/journal.pcbi.1004842.

71. Wakeley, J. (2009). Coalescent Theory: An Introduction (Roberts and Company Publishers).

72. Hadfield, J., Megill, C., Bell, S.M., Huddleston, J., Potter, B., Callender, C., Sagulenko, P., Bedford, T., and Neher, R.A. (2018). Nextstrain: real-time tracking of pathogen evolution. Bioinformatics *34*, 4121–4123, https://doi.org/10.1093/bioinformatics/bty407.

73. Kuhner, M.K., and Felsenstein, J. (1994). A simulation comparison of phylogeny algorithms under equal and unequal evolutionary rates. Mol. Biol. Evol. *11*, 459–468, https://doi.org/10.1093/oxfordjournals.molbev.a040126.

74. Steel, M.A., and Penny, D. (1993). Distributions of tree comparison metrics–some new results. Syst. Biol. *42*, 126–141, https://doi.org/10.1093/sysbio/42.2.126.

75. Bastola, A., Sah, R., Rodriguez-Morales, A.J., Lal, B.K., Jha, R., Ojha, H.C., Shrestha, B., Chu, D.K., Poon, L.L., Costello, A., et al. (2020). The first 2019 novel coronavirus case in Nepal. Lancet Infect. Dis. *20*, 279–280, https://doi.org/10.1016/S1473-3099(20)30067-0.

76. Boni, M.F., Lemey, P., Jiang, X., Lam, T.T.Y., Perry, B.W., Castoe, T.A., Rambaut, A., and Robertson, D.L. (2020). Evolutionary origins of the SARS-CoV-2 sarbecovirus lineage responsible for the covid-19 pandemic. Nat. Microbiol. *5*, 1408–1417, https://doi.org/10.1038/s41564-020-0771-4.

77. Li, X., Giorgi, E.E., Marichannegowda, M.H., Foley, B., Xiao, C., Kong, X.P., Chen, Y., Gnanakaran, S., Korber, B., and Gao, F. (2020). Emergence of SARS-CoV-2 through recombination and strong purifying selection. Sci. Adv. *6*, eabb9153, https://doi.org/10.1126/sciadv.abb9153.

78. Rambaut, A., Holmes, E.C., O'Toole, Á., Hill, V., McCrone, J.T., Ruis, C., du Plessis, L., and Pybus, O.G. (2020). A dynamic nomenclature proposal for SARS-CoV-2 lineages to assist genomic epidemiology. Nat. Microbiol. *5*, 1403–1407, https://doi.org/10.1038/s41564-020-0770-5.

79. O'Toole, A., McCrone, J., and Scher, E. (2020). pangolin 2.0. https://github.com/cov-lineages/pangolin.

80. Rambaut, A. (2009). Figtree v1. 3.1. http://tree.bio.ed.ac.uk/software/figtree/.

81. Argimón, S., Abudahab, K., Goater, R.J., Fedosejev, A., Bhai, J., Glasner, C., Feil, E.J., Holden, M.T., Yeats, C.A., Grundmann, H., et al. (2016). Microreact: visualizing and sharing data for genomic epidemiology and phylogeography. Microb. Genomics *2*, e000093, https://doi.org/10.1099/mgen.0.000093.

82. Tang, X., Wu, C., Li, X., Song, Y., Yao, X., Wu, X., Duan, Y., Zhang, H., Wang, Y., Qian, Z., et al. (2020). On the origin and continuing evolution of SARS-CoV-2. Natl. Sci. Rev. *7*, 1012–1023, https://doi.org/10.1093/nsr/nwaa036.

83. Zhang, X., Tan, Y., Ling, Y., Lu, G., Liu, F., Yi, Z., Jia, X., Wu, M., Shi, B., Xu, S., et al. (2020). Viral and host factors related to the clinical outcome of covid-19. Nature *583*, 437–440, https://doi.org/10.1038/s41586-020-2355-0.

84. Volz, E.M., Hill, V., McCrone, J.T., Price, A., Jorgensen, D., O'Toole, A., Southgate, J.A., Johnson, R., Jackson, B., Nascimento, F.F., et al. (2020). Evaluating the effects of SARS-CoV-2 spike mutation D614G on transmissibility and pathogenicity. Cell. https://doi.org/10.1016/j.cell.2020.11.020.

85. Korber, B., Fischer, W.M., Gnanakaran, S., Yoon, H., Theiler, J., Abfalterer, W., Hengartner, N., Giorgi, E.E., Bhattacharya, T., Foley, B., et al. (2020). Tracking changes in SARS-CoV-2 spike: evidence that d614g increases infectivity of the covid-19 virus. Cell *182*, 812–827, https://doi.org/10.1016/j.cell.2020.06.043.

86. Yurkovetskiy, L., Wang, X., Pascal, K.E., Tomkins-Tinch, C., Nyalile, T., Wang, Y., Baum, A., Diehl, W.E., Dauphin, A., Carbone, C., et al. (2020). Structural and functional analysis of the D614G SARS-CoV-2 spike protein variant. Cell *183*, 739–751, https://doi.org/10.1016/j.cell.2020.09.032.

87. Grubaugh, N.D., Hanage, W.P., and Rasmussen, A.L. (2020). Making sense of mutation: what D614G means for the COVID-19 pandemic remains unclear. Cell *182*, 794–795, https://doi.org/10.1016/j.cell.2020.06.040.

88. van Dorp, L., Richard, D., Tan, C.C., Shaw, L.P., Acman, M., and Balloux, F. (2020). No evidence for increased transmissibility from recurrent mutations in SARS-CoV-2. bioRxiv. https://doi.org/10.1101/2020.05.21.108506.

89. van Dorp, L., Acman, M., Richard, D., Shaw, L.P., Ford, C.E., Ormond, L., Owen, C.J., Pang, J., Tan, C.C., Boshier, F.A., et al. (2020). Emergence of genomic diversity and recurrent mutations in SARS-CoV-2. Infect. Genet. Evol. *83*, 104351, https://doi.org/10.1016/j.meegid.2020.104351.

90. Soares, P., Ermini, L., Thomson, N., Mormina, M., Rito, T., Röhl, A., Salas, A., Oppenheimer, S., Macaulay, V., and Richards, M.B. (2009). Correcting for purifying selection: an improved human mitochondrial molecular clock. Am. J. Hum. Genet. *84*, 740–759, https://doi.org/10.1016/j.ajhg.2009.05.001.

91. Letko, M., Marzi, A., and Munster, V. (2020). Functional assessment of cell entry and receptor usage for RNA and other lineage b betacoronaviruses. Nat. Microbiol. *5*, 562–569, https://doi.org/10.1038/s41564-020-0688-y.

92. Xia, S., Xu, W., Wang, Q., Wang, C., Hua, C., Li, W., Lu, L., and Jiang, S. (2018). Peptide-based membrane fusion inhibitors targeting hcov-229e spike protein HR1 and HR2 domains. Int. J. Mol. Sci. *19*, 487, https://doi.org/10.3390/ijms19020487.

93. Xia, S., Yan, L., Xu, W., Agrawal, A.S., Algaissi, A., Tseng, C.T.K., Wang, Q., Du, L., Tan, W., Wilson, I.A., et al. (2019). A pan-coronavirus fusion inhibitor targeting the hr1 domain of human coronavirus spike. Sci. Adv. *5*, https://doi.org/10.1126/sciadv.aav4580.

94. Li, X., Wang, W., Zhao, X., Zai, J., Zhao, Q., Li, Y., and Chaillon, A. (2020). Transmission dynamics and evolutionary history of 2019-nCoV. J. Med. Virol. *92*, 501–511, https://doi.org/10.1002/jmv.25701.

95. Fay, J.C., Wyckoff, G.J., and Wu, C.I. (2001). Positive and negative selection on the human genome. Genetics *158*, 1227–1234. https://www.genetics.org/content/158/3/1227.

96. Sobel Leonard, A., Weissman, D.B., Greenbaum, B., Ghedin, E., and Koelle, K. (2017). Transmission bottleneck size estimation from pathogen deep-sequencing data, with an application to human influenza a virus. J. Virol. *91*, https://doi.org/10.1128/JVI.00171-17.

97. Ghafari, M., Lumby, C.K., Weissman, D.B., and Illingworth, C.J. (2020). Inferring transmission bottleneck size from viral sequence data using a novel haplotype reconstruction method. J. Virol. https://doi.org/10.1128/JVI.00014-20.

98. Monsion, B., Froissart, R., Michalakis, Y., and Blanc, S. (2008). Large bottleneck size in cauliflower mosaic virus populations during host plant colonization. PLoS Pathog. *4*, 1–7, https://doi.org/10.1371/journal.ppat.1000174.

99. Lequime, S., Fontaine, A., Ar Gouilh, M., Moltini-Conclois, I., and Lambrechts, L. (2016). Genetic drift, purifying selection and vector genotype shape dengue virus intra-host genetic diversity in mosquitoes. PLoS Genet. *12*, 1–24, https://doi.org/10.1371/journal.pgen.1006111.

100. Gardy, J.L., and Loman, N.J. (2018). Towards a genomics-informed, real-time, global pathogen surveillance system. Nat. Rev. Genet. *19*, 9, https://doi.org/10.1038/nrg.2017.88.

101. Li, R., Pei, S., Chen, B., Song, Y., Zhang, T., Yang, W., and Shaman, J. (2020). Substantial undocumented infection facilitates the rapid dissemination of novel coronavirus (SARS-CoV-2). Science *368*, 489–493, https://doi.org/10.1126/science.abb3221.

102. Volz, E.M., Pond, S.L.K., Ward, M.J., Brown, A.J.L., and Frost, S.D. (2009). Phylodynamics of infectious disease epidemics. Genetics *183*, 1421–1430, https://doi.org/10.1534/genetics.109.106021.

103. Bedford, T., Greninger, A.L., Roychoudhury, P., Starita, L.M., Famulare, M., Huang, M.L., Nalla, A., Pepper, G., Reinhardt, A., Xie, H., et al. (2020). Cryptic transmission of SARS-CoV-2 in Washington state. Science *370*, 571–575, https://doi.org/10.1126/science.abc0523.

104. Gomez-Carballa, A., Bello, X., Pardo-Seco, J., Martinon-Torres, F., and Salas, A. (2020). Mapping genome variation of SARS-CoV-2 worldwide highlights the impact of COVID-19 super-spreaders. Genome Res. http://www.genome.org/cgi/doi/10.1101/gr.266221.120.

105. Ramazzotti, D., Angaroni, F., Maspero, D., Ascolani, G., Castiglioni, I., Piazza, R., Antoniotti, M., and Graudenzi, A. (2020). Longitudinal cancer evolution from single cells. bioRxiv. https://doi.org/10.1101/2020.01.14.906453.

106. Kimura, M. (1969). The number of heterozygous nucleotide sites maintained in a finite population due to steady flux of mutations. Genetics *61*, 893–903. https://www.genetics.org/content/61/4/893.

107. Gusfield, D. (1991). Efficient algorithms for inferring evolutionary trees. Networks *21*, 19–28, https://doi.org/10.1002/net.3230210104.

108. Huson, D.H., and Scornavacca, C. (2012). Dendroscope 3: an interactive tool for rooted phylogenetic trees and networks. Syst. Biol. *61*, 1061–1067, https://doi.org/10.1093/sysbio/sys062.

109. Mindell, D.P. (1993). Merger of taxa and the definition of monophyly (reply to Jan Zrzavỳ and Zdeněk Skála). Biosystems *31*, 130–133, https://doi.org/10.1016/0303-2647(93)90041-A.

110. Zrzavỳ, J., and Skála, Z. (1993). Holobionts, hybrids, and cladistic classification (reply to David P. Mindell). Biosystems *31*, 127–130, https://doi.org/10.1016/0303-2647(93)90040-J.

111. Chan, J.M., Carlsson, G., and Rabadan, R. (2013). Topology of viral evolution. Proc. Natl. Acad. Sci. U S A *110*, 18566–18571, https://doi.org/10.1073/pnas.1313480110.

112. Skála, Z., and Zrzavỳ, J. (1994). Phylogenetic reticulations and cladistics: discussion of methodological concepts. Cladistics *10*, 305–313, https://doi.org/10.1111/j.1096-0031.1994.tb00180.x.

113. Brandstätter, A., Sänger, T., Lutz-Bonengel, S., Parson, W., Béraud-Colomb, E., Wen, B., Kong, Q.P., Bravi, C.M., and Bandelt, H.J. (2005). Phantom mutation hotspots in human mitochondrial DNA. Electrophoresis *26*, 3414–3429, https://doi.org/10.1002/elps.200500307.

114. Weissensteiner, H., Pacher, D., Kloss-Brandstätter, A., Forer, L., Specht, G., Bandelt, H.J., Kronenberg, F., Salas, A., and Schönherr, S. (2016). HaploGrep 2: mitochondrial haplogroup classification in the era of high-throughput sequencing. Nucleic Acids Res. *44* (W1), W58–W63, https://doi.org/10.1093/nar/gkw233.

115. Srinivas, G., Möller, S., Wang, J., Künzel, S., Zillikens, D., Baines, J.F., and Ibrahim, S.M. (2013). Genome-wide mapping of gene–microbiota

CellPress
OPEN ACCESS

interactions in susceptibility to autoimmune skin blistering. Nat. Commun. *4*, 2462, https://doi.org/10.1038/ncomms3462.

116. Pearl, J. (2009). Causality (Cambridge University Press).

117. Luecken, M.D., and Theis, F.J. (2019). Current best practices in single-cell RNA-seq analysis: a tutorial. Mol. Syst. Biol. *15*, https://doi.org/10.15252/msb.20188746.

118. Traag, V.A., Waltman, L., and van Eck, N.J. (2019). From Louvain to Leiden: guaranteeing well-connected communities. Sci. Rep. *9*, 1–12, https://doi.org/10.1038/s41598-019-41695-z.

119. Koboldt, D.C., Zhang, Q., Larson, D.E., Shen, D., McLellan, M.D., Lin, L., Miller, C.A., Mardis, E.R., Ding, L., and Wilson, R.K. (2012). Varscan 2: Somatic mutation and copy number alteration discovery in cancer by exome sequencing. Genome Res. *22*, 568–576, https://doi.org/10.1101/gr.129684.111.

120. De Maio, N., Walker, C., Borge, R., Weilguny, L., Slodkowick, G., and Goldmand, N. (2020). Issues with SARS-CoV-2 sequencing data. https://virological.org/.

121. Park, D., Tomkins-Tinch, C., Ye, S., Jungreis, I., Shlyakhter, I., Metsky, H., Hanna, Lin, M., Le, V., Lin, A., et al. (2019). broadinstitute/viral-ngs: v1.25.0. https://doi.org/10.5281/zenodo.3509008.

122. Bar-On, Y.M., Flamholz, A., Phillips, R., and Milo, R. (2020). Science forum: SARS-CoV-2 (COVID-19) by the numbers. eLife *9*, e57309, https://doi.org/10.7554/eLife.57309.

Patterns, Volume *2*

Supplemental information

VERSO: A comprehensive framework for the inference of robust phylogenies and the quantification of intra-host genomic diversity of viral samples

Daniele Ramazzotti, Fabrizio Angaroni, Davide Maspero, Carlo Gambacorti-Passerini, Marco Antoniotti, Alex Graudenzi, and Rocco Piazza

Contents

1 Additional materials and methods

1.1 Additional details on VERSO STEP #1 algorithmic framework

1.2 Performance assessment metrics

1.3 Parameter settings

1.4 Summary of notation

2 Additional results

2.1 Reference genome generation

2.2 Expanded clonal variants tree – Dataset #1

2.3 Mutational hotspot analysis – Dataset #1

2.4 Bottleneck size estimation – Dataset #1

2.5 Impact of clonal variant threshold on VERSO stability – Dataset #1

2.6 Application of VERSO to 1438 samples from RNA sequencing data (Dataset #2)

2.7 Expanded clonal variants tree – Dataset #2

2.8 Computation time

1 Additional materials and methods

1.1 Additional details on VERSO STEP #1 algorithmic framework

VERSO STEP #1 aims at inferring robust phylogenomic models from either raw sequencing data or consensus sequences, and employs an algorithmic strategy akin to[1]. In particular, VERSO STEP #1 takes as input either: (i) (if raw sequencing data are available) the binarized profiles of clonal SNVs detected in the cohort, which are selected via a variant frequency (VF) threshold (equal to 90% in the case studies) or, (ii) the consensus sequences of the samples in the cohort, which are translated in opportune binarized clonal variants profiles by employing a reference genome (see the main text).

More in detail, let us consider k different clonal genotypes characterized by m clonal variants and n samples $s_1, \ldots, s_n$. We define the *theoretical clonal genotype matrix* $\mathbf{G}$: a binary $n \times m$ matrix where each row represents a sample and each column a clonal mutation; an element of $\mathbf{G}$, $g_{i,j} = 1$ if variant j is present in sample i, otherwise $g_{i,j} = 0$. We then define the *phylogenetic matrix* $\mathbf{B}$: a binary $k \times m$ matrix where each row represents a clonal genotype and each column a clonal mutation; $b_{ij} = 1$ if we detect mutation j in clonal genotype i, otherwise $b_{i,j} = 0$. Each $\mathbf{B}$ uniquely identifies a clonal variants tree[2]. We here assume the existence of a perfect phylogeny process and that the Infinite Sites Assumption holds[3], i.e., mutations cannot be lost, they can compare only once in the tree, and the tree has only one root. Notice that violations of the Infinite Sites Assumption are possible due to, e.g., homoplasies or loss of mutations, which are characterized after the inference (see the specific features on homoplasy detection described in the main text).

Finally, we define the *sample attachment matrix* $\mathbf{S}$: a binary $n \times k$ matrix, where each row represents a sample and each column a clonal genotype; $s_{i,j} = 1$ if sample i is associate to clonal genotype j, otherwise $s_{i,j} = 0$; each sample is attached exactly to one clonal genotype.

Therefore, the following factorization holds:

$$\mathbf{G} = \mathbf{S} \cdot \mathbf{B}. \tag{1}$$

In general, the observed clonal genotype of a given sample i in real-world data may not correspond to its theoretical clonal genotype (the i-th row of $\mathbf{G}$), because it can include include false positives, false negatives and missing values. Therefore, when considering a real-word data matrix ($\mathbf{D}$), the previous equation typically does not hold. For this reason, VERSO employs a maximum likelihood estimation procedure to determine the model that best fits the data, given an appropriate noise model. Let us define the data matrix $\mathbf{D}$ a binary $n \times m$ matrix where each row represents a sample and each column a clonal mutation; an element of $\mathbf{D}$, $d_{i,j} = 1$ if the variant j is present in sample i, otherwise $d_{i,j} = 0$. Then, the posterior probability of an output model (i.e., $\mathbf{B}$, $\mathbf{S}$), and of error rates α (i.e., the false positive rate) and β

(i.e., the false negative rate), given the data matrix $\mathbf{D}$ and the hyperparameters of error rate distributions θ_α and θ_β can be written as:

$$P(\mathbf{B},\mathbf{S},\mathbf{G},\alpha,\beta|\mathbf{D},\theta_\alpha,\theta_\beta) \propto P(\mathbf{D}|\mathbf{B},\mathbf{S},\mathbf{G},\alpha,\beta,\theta_\alpha,\theta_\beta)P(\mathbf{B},\mathbf{S},\mathbf{G},\alpha,\beta|\theta_\alpha,\theta_\beta). \tag{2}$$

We then assume independence of both the phylogenetic model $\mathbf{B}$ and the sample attachment $\mathbf{S}$ from error rates and a uniform uninformative prior on θ_α and θ_β. Furthermore, we assume that the data matrix $\mathbf{D}$ depends only on theoretical clonal genotype matrix $\mathbf{G}$ and error rates α and β, we obtain:

$$P(\mathbf{B},\mathbf{S},\mathbf{G},\alpha,\beta|\mathbf{D},\theta_\alpha,\theta_\beta) \propto P(\mathbf{D}|\mathbf{G},\alpha,\beta)P(\mathbf{G}|\mathbf{B},\mathbf{S})P(\mathbf{S}|\mathbf{B})P(\mathbf{B})P(\alpha|\theta_\alpha)P(\beta|\theta_\beta). \tag{3}$$

Finally, assuming: *i)* an uniform uninformative prior on $\mathbf{B}$, *ii)* that given the hyper-parameters of error rates, all possible values of α and β are equiprobable (this because, in the current formulation, VERSO includes a noise estimation procedure via grid search over a limited number of values of α and β), and *iii)* that given any $\mathbf{B}$, all $\mathbf{S}$ are equiprobable, we obtain:

$$P(\mathbf{B},\mathbf{S},\mathbf{G},\alpha,\beta|\mathbf{D},\theta_\alpha,\theta_\beta) \propto P(\mathbf{D}|\mathbf{G},\alpha,\beta). \tag{4}$$

Then, the problem is reduced to the maximization of the following likelihood function:

$$P(\mathbf{B},\mathbf{S},\mathbf{G},\alpha,\beta|\mathbf{D},\theta_\alpha,\theta_\beta) \propto \prod_{i=1}^{n}\prod_{j=1}^{m} P(d_{i,j}|\mathbf{G}_{i,j},\alpha,\beta), \tag{5}$$

where $d_{i,j}$ is a entry of $\mathbf{D}$ and:

$$P(d_{i,j}|\mathbf{G}_{i,j},\alpha,\beta) = \begin{cases} \alpha, & if \quad d_{i,j}=1 \quad and \quad \mathbf{G}_{i,j}=0, \\ 1-\alpha, & if \quad d_{i,j}=1 \quad and \quad \mathbf{G}_{i,j}=1, \\ \beta, & if \quad d_{i,j}=0 \quad and \quad \mathbf{G}_{i,j}=1, \\ 1-\beta, & if \quad d_{i,j}=0 \quad and \quad \mathbf{G}_{i,j}=0, \\ 1, & if \quad d_{i,j}=\mathrm{NA}, \end{cases} \tag{6}$$

where NA label missing entries, e.g., due to low coverage. In Figure S1 the graphical probabilistic model of VERSO is shown .

As an exhaustive search can be performed only for very small models, we employ a Markov Chain Monte Carlo (MCMC) scheme via a Metropolis–Hastings algorithm, to search the model that maximizes the likelihood function defined in (5) . The MCMC includes two ergodic moves on $\mathbf{B}$: (i) node relabeling, (ii) prune/reattach of a single node and its descendants. For each proposed configuration $\mathbf{B}'$, we find the maximum likelihood $\mathbf{S}'$ via an exhaustive search and we then evaluate the new proposed corrected clonal genotype $\mathbf{G}'$. Therefore, the acceptance ratio of a move $\rho_{\mathbf{B}',\mathbf{S}',\mathbf{G},\alpha,\beta}$ is given by:

$$\rho_{\mathbf{B}',\mathbf{S}',\mathbf{G},\alpha,\beta} = \min\left\{ \left(\frac{P(\mathbf{D}|\mathbf{G}',\alpha,\beta)}{P(\mathbf{D}|\mathbf{G},\alpha,\beta)}\right)^{1/T}, 1 \right\}, \tag{7}$$

where T is a learning rate parameter, which can be tuned as suggested in[4] (default $T = 1$).

Once the maximum likelihood model ($\mathbf{B}$, $\mathbf{S}$, α and β) is found, the clonal variants tree $\mathbf{B}$ represents the inner structure of the output phylogenetic tree in which variants are the inner nodes, whereas the sample attachment matrix $\mathbf{S}$ determines the edges connecting the inner nodes to the samples, which are the leaves of the phylogenetic tree. Since the number of corrected clonal genotypes is typically much lower than the number of samples, samples with the same corrected genotype are grouped in polytomies, i.e., they are children of the same inner node.

Notice that, in the output phylogenetic tree, the branch length correspond to the number of clonal substititions, which can be normalized according to the genome length. Finally, in addition to the maximum likelihood phylogenetic model, VERSO also provides as output the corrected clonal genotype of all samples.

1.2 Performance assessment metrics

The performance of the different methods was assessed by simulations, comparing the inferred phylogenies with the respective ground-truth topology; three metrics were considered (i) absolute error evolutionary distance, (ii) branch score difference[5] and (iii) quadratic path difference[6], which are detailed in the following.

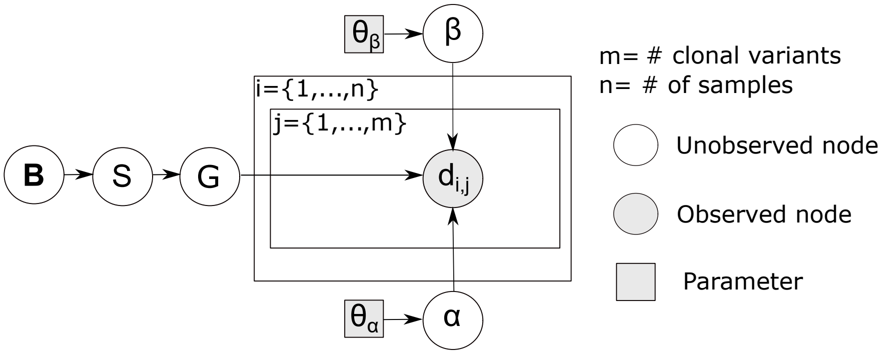

Figure S1: **Probabilistic graphical model of VERSO STEP #1.** Shaded nodes represent observed values. Square and shaded nodes represent fixed parameters; unshaded nodes represent latent variables. The probability distribution of unshaded nodes is approximated using samples from the proposed search scheme. Please refer to the text for the definition of variables and parameters.

(i) **Absolute Error Evolutionary Distance.** For two phylogenetic trees, we compute the pairwise distances between each pairs of tips using its branch lengths. The Absolute Error Evolutionary Distance is the mean absolute error between such distances in the two trees.

(ii) **Branch Score Difference.** To compute this distance, we consider the list of all possible partitions over two phylogenetic trees, some of which will be found in the trees, while others correspond to branches that are not admissible in some of them; branch lengths of 0 are assigned to these latter partitions. For the two trees, the Branch Score Difference[5] is the sum of squared differences between such branch lengths.

(iii) **Quadratic Path Difference.** The length of a path connecting one tip to another in a phylogenetic tree is the number of edges that must be navigated to get from the first tip to the other. Given two phylogenetic trees, the Quadratic Path Difference[6] is the square root of the sum of squares of the difference in path length between each pair of tips.

1.3 Parameter settings

**Parameters of simulations via msprime[7]**				
**# of simulations**	20			
**Sample size n**	1000			
**Effective population size**	0.5			
**Mutational rate**	2×10^{-6} mutations per generation			
**Genome length**	29903			
**Features of the sythetic datasets**				
**Scenario**	A	B	C	D
**# of datasets**	20	20	20	20
**# of Samples**	1000	1000	500	500
false positive rate α	0.05	0.1	0.05	0.1
false negative rate β	0.05	0.1	0.05	0.1

Table S1: Parameter settings of the coalescent simulations via msprime[7] and of the related datasets employed in the comparative assessment presented in the main text.

Settings of phylogenetic methods on simulated datasets	
**VERSO STEP $\#1$**	
**MCMC iterations**	100000
**# of MCMC restarts**	500
**Early stop threshold**	5000
**Learning rate T**	1
**Grid search α**	$[0.001, 0.010, 0.050, 0.100]$
**Grid search β**	$[0.001, 0.010, 0.050, 0.100]$
**IQ-TREE** - Launched via Nextstrain-Augur pipeline	
**Software version**	1.6.12
**Substitution model**	GTR
**Other parameters**	Default
**BEAST 2**	
**Software version**	2.6.3
**MCMC iterations**	1×10^6
**Substitution model**	GTR
**Clock rate**	fixed (=1)
**Population model**	costant (=1)
**All priors**	default
**All move probability**	default
**Burn-in of trees**	10%
**Target tree type**	maximun clade credibility
**Node heights**	median

Table S2: Parameters settings of VERSO STEP $\#1$, IQ-TREE and Beast 2 with respect to the comparative assessment on simulations presented in the main text. IQ-TREE was launched via Nextstrain-Augur pipeline[8].

**Features of real-world datasets**		
**Dataset**	Dataset #1	Dataset #2
**Sequencing protocol**	Amplicon	RNA-seq
**# of samples - pre QC**	3960	2766
**# of samples - post QC**	2906	1438
**# of variants - pre QC**	55280	53354
**# of variants - post QC**	10571	6143
**# of clonal variants - included in the inference**	29	23
**Clonal threshold** δ	0.9	0.9
**VERSO STEP #1: parameter settings**		
**MCMC iterations**	100000	100000
**# of MCMC restarts**	500	500
**Early stop threshold**	5000	5000
**Learning rate** T	1	1
**Grid search** α	$[0.001, 0.010, 0.050, 0.100]$	$[0.001, 0.010, 0.050, 0.100]$
**Grid search** β	$[0.001, 0.010, 0.050, 0.100]$	$[0.001, 0.010, 0.050, 0.100]$
**VERSO STEP #2: parameter settings**		
**Distance type**	Bray-Curtis diss.	Bray-Curtis diss.
**# of PCAs**	10	10
**# of** k **(kNN)**	10	10
**Leiden resolution**	1	1
**Spread (UMAP)**	1	1
**Min_dist (UMAP)**	2	2
**a (UMAP)**	0.01	0.01
**b (UMAP)**	1	1

Table S3: Features of the real-world datasets employed in the case studies and parameter settings of both steps of VERSO with respect to the application of such datasets.

1.4 Summary of notation

Symbol	Description
m	Number of clonal variants
k	Number of clonal genotypes in the model
n	Number of samples
α	False Positive Rate
β	False Negative Rate
γ	Ratio of missing entries
δ	Clonal variant VF threshold
$\mathbf{G}$	Theoretical clonal genotype matrix
$\mathbf{B}$	Perfect phylogeny matrix
$\mathbf{D}_i$	Input binary clonal variant profile matrix
$\mathbf{S}$	Sample attachment matrix
$b_{i,j}$	i, j element of the matrix $\mathbf{B}$
$d_{i,j}$	i, j element of the matrix $\mathbf{D}$
v_i	ordered vector of the VF of the i-th sample
$s_{i,j}$	i, j element of the matrix $\mathbf{S}$
$\mathbf{b}_i$	i-th row of the phylogenetic matrix $\mathbf{B}$
$\rho_{\mathbf{B}',\mathbf{S}',\mathbf{G},\alpha,\beta}$	Ratio of acceptance of a (i.e., the proposal of a $\mathbf{B}'$ and $\mathbf{C}'$) in the MCMC
T	Learning rate parameter in the MCMC
$d(a, b)$	Bray-Curtis dissimilarity between a and b
TP	True positive
FP	False positive
TN	True negative
FN	False negative
VF_i^j	Variant frequency of the j-th sample in the i-th nucleotide

Table S4: Summary of the main notation used in the article.

2 Additional results

2.1 Reference genome generation

The description of the generation of reference genome SARS-CoV-2-ANC is provided in the main text and depicted in Figure S2.

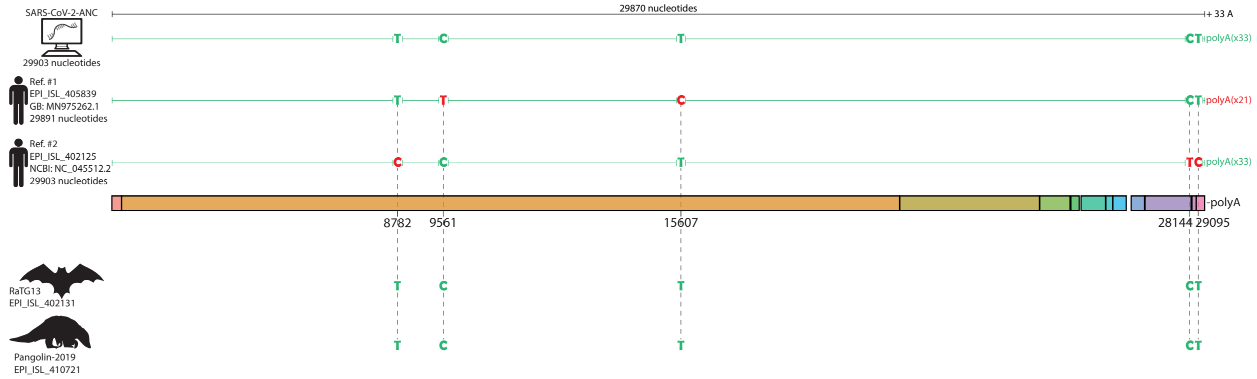

Figure S2: **Generation of SARS-CoV-2-ANC reference genome.** The artificial reference genome SARS-CoV-2-ANC was generated setting 29865 (on 29870) genome locations identical to both ref #1 (EPI_ISL_405839) and ref #2 (EPI_ISL_402125), including the polyA tail of ref. #2 (33 bases), and setting haplotype TCTCT at locations 8782, 9561, 15607, 28144 and 29095, which is shared by both the Bat-CoV-RaTG13 genome (sequence EPI_ISL_402131) and the Pangolin-CoV genome (sequence EPI_ISL_410721). The final length of the SARS-CoV-2-ANC genome is 29903.

2.2 Expanded clonal variants tree – Dataset #1

In Figure S3 we report the *expanded* clonal variants tree of Dataset #1, obtained by duplicating homoplastic variants identified after the phylogenetic tree inference via VERSO STEP #1. In particular, the 5 homoplastic variants were duplicated in the tree, if observed in at least 10 samples with the same corrected clonal genotype, and were positioned after the corresponding characterizing mutation, as proposed in[9]. Homoplastic variants were finally connected via dashed lines to pinpoint reticulation events. In Figure 3 of the main text, all samples displaying homoplastic variants are highlighted directly on the phylogenetic model returned by VERSO STEP #1.

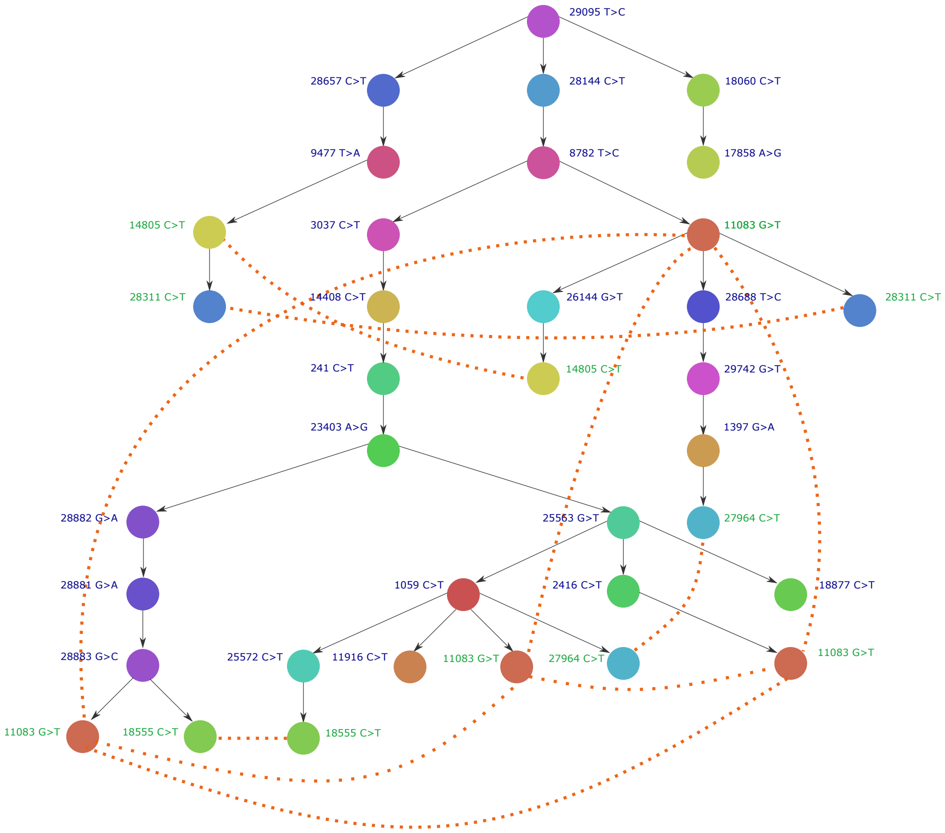

Figure S3: **Expanded clonal variants tree – Dataset #1.** The clonal variants tree **B** representing the inner structure of the output phylogenetic tree inferred from Dataset #1 via VERSO STEP #1 is expanded by duplicating homoplastic variants. Nodes represent clonal variants (consistent colors with Figure 3 of the Main Text), whereas edges indicate temporal ordering/accumulation relations. Homoplastic mutations were duplicated if observed in at least 10 samples with the same corrected clonal genotype returned by VERSO STEP #1 and are attached after the corresponding characterizing mutation. Homoplastic variants observed in multiple samples with the same corrected clonal genotype are ordered by the number of samples in which they are detected. Dashed lines connect identical homoplastic mutations.

2.3 Mutational hotspot analysis – Dataset #1

To identify the regions of the SARS-CoV-2 genome more prone to mutations, which might be due to *natural* mutational hotspots or to *phantom mutations*, i.e., systematic artifacts generated during sequencing processes[10], it is sound to evaluate the mutation density with a sliding-window approach, as proposed, for instance, in[11]. This analysis is also effective to distinguish homoplasies due to positive selection from those due to artifacts or hotspots.

More in detail, the analysis considers: (i) minor variants (i.e., with VF$< 90\%$) to prevent possible biases due to the transmission and accumulation of clonal variants in the population, (ii) with synonymous substitutions, to remove positively selected variants. Here, we considered a 300-base window, which was iteratively slid along the genome 1 base at a time. For every iteration and for each window, we first computed the total number of distinct synonymous SNVs fall within the window, and normalized with respect to the window length. Finally, we associate a density value to every genome location, that is the mean value of all windows which include such location.

We executed the analysis on Dataset $\#1$ and we further investigated the properties of: (i) the 5 clonal variants identified as possible homoplasies via VERSO STEP $\#1$, (ii) 80 selected minor variants: (1) detected in multiple clonal genotypes (identified via VERSO STEP $\#1$), (2) detected in at least 10 samples, (3) nonsynonymous, (4) falling on a region of the genome with mutational density lower than the median value ($= 0.083$ [syn. mutations][nucleotides]$^{-1}$) (4 mutations were filtered-out after manual curation).

In Figure S4 the mutational density of the SARS-CoV-2 genome (Fig. S4**A**) and the related empirical distribution (Fig. S4**B**) are shown, as computed from the 2906 samples of Dataset $\#1$, by highlighting the clonal variants mentioned above. Furthermore, Tables S5 and S6 include summary features for all such variants.

Importantly, this analysis allows to assess whether a homoplasy detected via VERSO might have been positively selected due to some functional advantage or, instead, if it may be due to mutational hotspots or phantom mutations. The results on Dataset $\#1$ are discussed in the main text.

Homoplasies: clonal variants

**Variant**	**Genome location**	**Variant effect**	**Amino Acid substitution**	**# of samples**	**Mutational density**
11083 G>T	ORF1ab	NS	3606 L>F	460	0.085
27964 C>T	ORF8	NS	24 S>L	182	0.124
28311 C>T	N	NS	13 P>L	153	0.100
14805 C>T	ORF1ab	S	-	237	0.082
18555 C>T	ORF1ab	S	-	152	0.103

Table S5: **Summary features of candidate homoplastic clonal variants.** The table includes the main features of the 5 clonal variants identified as candidate homoplasies via VERSO STEP $\#1$.

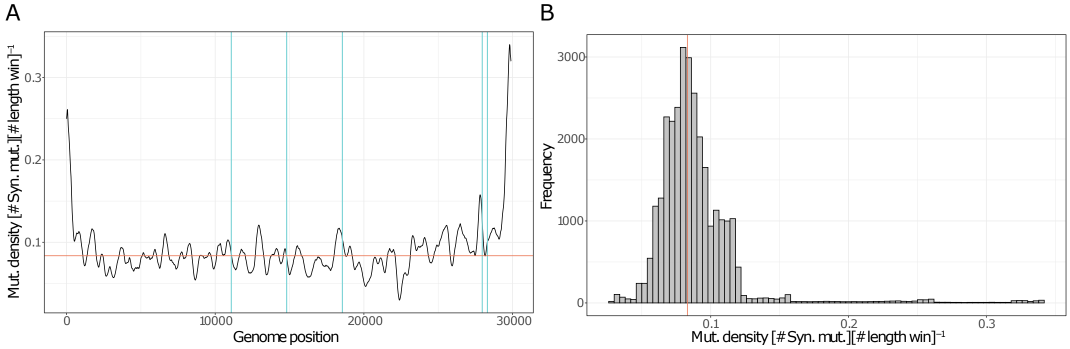

Figure S4: **Characterization of the mutational density over the SARS-CoV-2 genome.** **(A)** The mutational density, evaluated on synonymous minor variants detected in the samples of Dataset $\#1$ via the sliding-window approach described in the text, is projected on the SARS-CoV-2 genome. The vertical lines represent the positions of 5 clonal variants involved in homoplasies and discussed in the main text (light blue) (the horizontal solid line represent the median mutational density). **(B)** The related empirical distribution is shown.

2.4 Bottleneck size estimation – Dataset $\#1$

To have an estimation of the size of the genetic bottleneck in SARS-CoV-2 we evaluate how the variance of some selected SNVs detected in the samples of Dataset $\#1$ changes upon time[12,13]. In principle, if the evolution is purely neutral, the changes in the genetic variance between two different time points are exclusively due to transmission bottleneck effects.

We first divided the time interval under study (i.e., 30 weeks, from the 1-st week of the 2020 to 29-th week of 2020) into two equal intervals (i.e., $t_1 \leq 14$-th week or $t_2 > 14$-th week).

Then, in order to minimize the effect of the genetic drift and to consider comparison between groups in which a transmission event might have occurred, we restrict the analysis to group of samples characterized by distinct clonal genotypes identified via VERSO STEP $\#1$. It is also sound to select a number of SNVs that are presumably neutral or quasi-neutral markers, as proposed in[12,13]. To this end, we employed the following criteria: *i)* we take into account only

Variant	# of clonal genotypes	# of samples	Type	Genomic location	Amino Acid substitution	Mutational density	Average VF
5743 G>C	15	96	alway minor	ORF1ab	1826E>D	0.08	0.159
5744 T>C	15	95	alway minor	ORF1ab	1827Y>H	0.08	0.178
17747 C>T	4	78	mixed	ORF1ab	5828P>L	0.066	0.601
2192 A>G	2	51	alway minor	ORF1ab	643R>G	0.071	0.094
12164 G>T	12	50	alway minor	ORF1ab	3967A>S	0.069	0.181
2578 T>A	6	48	alway minor	ORF1ab	771S>R	0.065	0.095
24557 G>T	12	47	alway minor	S	999G>C	0.075	0.141
8790 G>T	11	47	alway minor	ORF1ab	2842G>V	0.064	0.167
11535 G>T	12	46	alway minor	ORF1ab	3757G>V	0.076	0.15
17096 G>T	12	46	alway minor	ORF1ab	5611G>V	0.079	0.112
7038 G>T	11	45	alway minor	ORF1ab	2258G>V	0.078	0.184
2036 G>T	13	43	alway minor	ORF1ab	591A>S	0.078	0.143
9249 G>T	11	43	alway minor	ORF1ab	2995G>V	0.08	0.135
13571 G>T	12	40	alway minor	ORF1ab	4436G>V	0.065	0.122
5766 G>C	12	40	alway minor	ORF1ab	1834G>A	0.081	0.11
20135 T>A	4	40	mixed	ORF1ab	6624V>E	0.046	0.47
14906 A>C	3	40	alway minor	ORF1ab	4881N>T	0.069	0.068
5765 G>A	12	39	alway minor	ORF1ab	1834G>S	0.081	0.11
4505 G>T	11	39	alway minor	ORF1ab	1414G>C	0.065	0.122
2604 G>T	11	38	alway minor	ORF1ab	780G>V	0.062	0.115
7214 G>T	11	34	alway minor	ORF1ab	2317D>Y	0.073	0.085
22899 G>T	10	34	alway minor	S	446G>V	0.06	0.152
9141 G>T	10	33	alway minor	ORF1ab	2959G>V	0.083	0.152
17848 G>T	9	33	alway minor	ORF1ab	5862G>C	0.069	0.088
16787 G>T	11	32	alway minor	ORF1ab	5508G>V	0.078	0.07
7246 G>T	10	32	alway minor	ORF1ab	2327W>C	0.073	0.103
15890 C>A	4	28	alway minor	ORF1ab	5209T>K	0.081	0.324
5375 G>T	9	26	alway minor	ORF1ab	1704A>S	0.081	0.116
7017 G>T	9	25	alway minor	ORF1ab	2251G>V	0.078	0.106
20465 A>G	4	25	mixed	ORF1ab	6734D>G	0.058	0.516
20387 A>G	3	25	mixed	ORF1ab	6708K>R	0.055	0.248
17849 G>T	10	24	alway minor	ORF1ab	5862G>V	0.069	0.077
14536 C>T	7	24	alway minor	ORF1ab	4758L>F	0.078	0.069
16514 A>G	2	24	mixed	ORF1ab	5417Y>C	0.064	0.24
3004 G>T	10	23	alway minor	ORF1ab	913E>D	0.064	0.118
8696 G>T	10	22	alway minor	ORF1ab	2811A>S	0.055	0.072
2247 G>T	9	22	alway minor	ORF1ab	661G>V	0.076	0.08
3096 C>T	12	21	mixed	ORF1ab	944S>L	0.058	0.204
16188 G>T	10	21	alway minor	ORF1ab	5308W>C	0.059	0.067
3259 G>T	9	21	alway minor	ORF1ab	998Q>H	0.061	0.091
17380 T>C	3	21	mixed	ORF1ab	5706Y>H	0.07	0.28
16516 A>G	2	21	mixed	ORF1ab	5418K>E	0.065	0.252
7363 G>T	10	20	alway minor	ORF1ab	2366W>C	0.067	0.073
11556 A>T	3	20	alway minor	ORF1ab	3764E>V	0.078	0.083
17929 A>C	2	20	alway minor	ORF1ab	5889I>L	0.077	0.286
9223 C>A	2	20	alway minor	ORF1ab	2986H>Q	0.082	0.224
9843 T>A	4	19	alway minor	ORF1ab	3193L>*	0.07	0.122
17933 C>G	2	19	alway minor	ORF1ab	5890T>S	0.077	0.241
12141 C>T	10	18	alway minor	ORF1ab	3959T>I	0.071	0.083
5617 G>T	9	18	alway minor	ORF1ab	1784Q>H	0.079	0.068
15202 G>T	9	18	alway minor	ORF1ab	4980V>L	0.071	0.067
21137 A>G	8	18	mixed	ORF1ab	6958K>R	0.081	0.227
15157 C>A	8	18	alway minor	ORF1ab	4965Q>K	0.069	0.191
17663 A>G	2	18	alway minor	ORF1ab	5800Y>C	0.069	0.157
19252 G>T	7	17	alway minor	ORF1ab	6330V>L	0.079	0.078
16376 C>T	4	16	mixed	ORF1ab	5371P>L	0.058	0.327
11562 G>A	2	16	alway minor	ORF1ab	3766C>Y	0.079	0.193
4917 T>A	2	16	mixed	ORF1ab	1551I>N	0.071	0.147
16508 G>T	8	15	alway minor	ORF1ab	5415G>V	0.064	0.081
2891 G>A	2	15	mixed	ORF1ab	876A>T	0.07	0.635
5658 T>A	2	15	mixed	ORF1ab	1798V>E	0.08	0.221
15199 G>T	8	14	alway minor	ORF1ab	4979V>L	0.071	0.067
5378 G>T	8	14	alway minor	ORF1ab	1705G>C	0.081	0.061
3896 G>T	7	14	alway minor	ORF1ab	1211V>F	0.082	0.063
12053 C>G	2	14	alway minor	ORF1ab	3930L>V	0.08	0.197
12041 G>T	8	13	alway minor	ORF1ab	3926D>Y	0.081	0.068
2250 G>T	8	13	alway minor	ORF1ab	662G>V	0.077	0.067
4854 G>T	7	13	alway minor	ORF1ab	1530R>I	0.068	0.092
4746 C>T	2	13	alway minor	ORF1ab	1494S>F	0.068	0.144
17365 T>C	2	13	alway minor	ORF1ab	5701S>P	0.071	0.112
16068 G>T	8	12	alway minor	ORF1ab	5268E>D	0.064	0.081
19679 G>T	6	12	alway minor	ORF1ab	6472G>V	0.076	0.075
21301 C>T	2	12	alway minor	ORF1ab	7013P>S	0.07	0.115
25913 G>C	5	11	alway minor	ORF3a	174G>A	0.072	0.111
8688 G>T	5	11	alway minor	ORF1ab	2808G>V	0.055	0.082
11288 T>G	2	11	alway minor	ORF1ab	3675S>A	0.069	0.13
16417 A>G	2	11	alway minor	ORF1ab	5385T>A	0.059	0.098
13627 G>T	7	10	mixed	ORF1ab	4455D>Y	0.073	0.219
12079 G>T	3	10	mixed	ORF1ab	3938R>S	0.078	0.183
24552 T>C	2	10	alway minor	S	997I>T	0.075	0.092

Table S6: **Summary features of candidate homoplastic minor variants.** The table includes the main features of the 80 minor variants identified as candidate homoplasies, ordered by number of samples in which they are detected (see the main text).

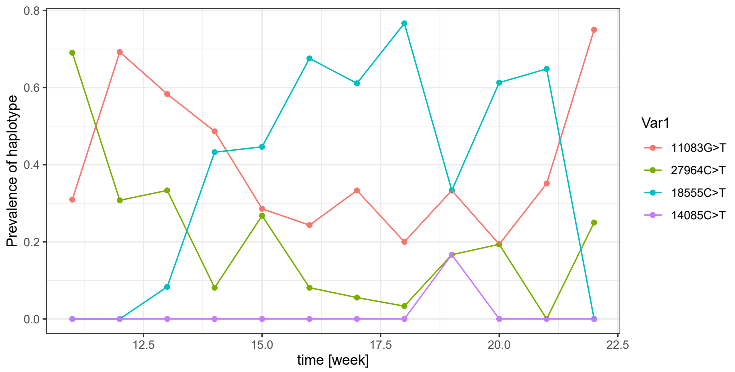

Figure S5: **Dynamics of the haplotypes related to 4 candidate homoplastic clonal variants.** Temporal evolution of prevalence of 4 haplotypes associated to homoplastic clonal variants discussed in the main text. The prevalence of the SNV g.28311C$>$T is not shown, because related samples have no collection date.

always minor variants (i.e., variants with VF $< 90\%$ in all samples in which they are detected), *ii)* the SNV must either be a synonymous change at the third codon position or fall in a non-coding region, *(iii)* the SNV must be observed in at least 3 samples in each time interval and, *iv)* no significant changes in mean VF of the SNV must be observed between the two time intervals (i.e., ≤ 0.10). Notice that the analysis is performed on groups of samples with distinct clonal genotypes, separately.

Applying these filters, we identified 3 distinct SNVs as quasi-neutral markers for Dataset $\#1$ (see Table S7 for the complete list of markers), two them (g.634T$>$C and g.14523A$>$G) are detected in genotype G15, while g.15168G$>$A is detected in genotypes G17 and G21. Note that only the samples with collection date were used in the analysis.

In Figure S6, the distribution of the VF of all markers, divided by time point and clonal genotype is presented. From this analysis, the presence of mild bottleneck effects is highlighted, as confirmed in other study considering the donor-host data[14].

Mutation id.	VF Mean at t_1	VF Mean at t_2	N. Obs. t_1	N. Obs. t_2	Genotype
g.634T>C	0.141 ± 0.005	0.082 ± 0.001	10	10	G15
g.14523A>G	0.253 ± 0.017	0.235 ± 0.015	14	17	G15
g.15168G>A	0.10 ± 0.0005	0.071 ± 0.001	4	4	G17
g.15168G>A	0.152 ± 0.003	0.091 ± 0.00005	25	5	G21

Table S7: **Summary features of the three SNVs selected as neutral markers employed in the bottleneck size estimation analysis.**

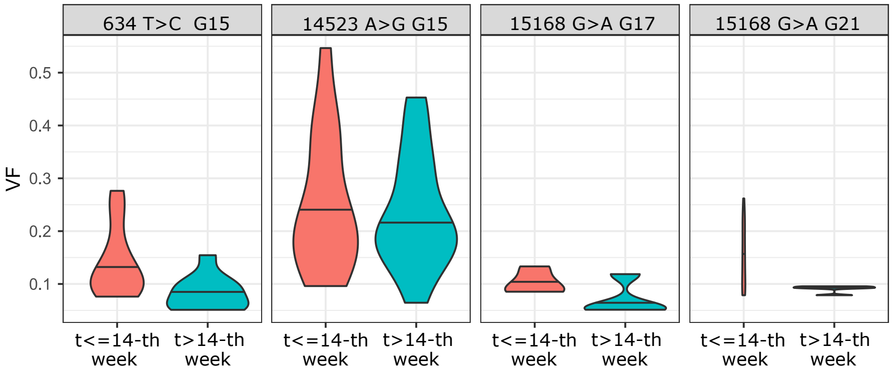

Figure S6: **Bottleneck size estimation** . For each SNV selected as neutral or quasi neutral marker (see Table S7) we show the violin plot of the VF distribution with respect to each clonal genotype, in the subsequent time intervals $t \leq 14$ and $t > 14$.

2.5 Impact of clonal variant threshold on VERSO stability – Dataset $\#1$

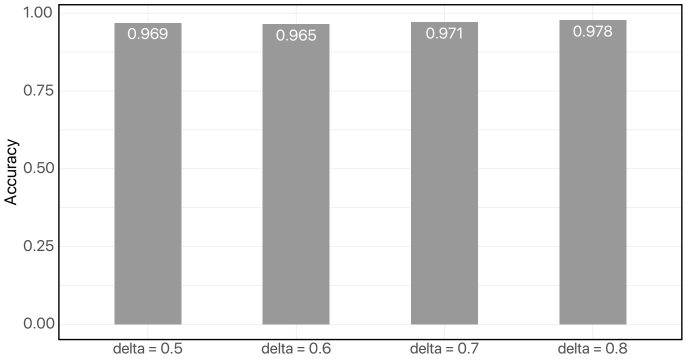

Figure S7: **Stability of VERSO to clonal variant threshold.** Variation of the tree accuracy obtained by changing the threshold used to define a clonal variants (δ), by setting as ground-truth the model returned by VERSO STEP $\#1$ with $\delta = 0.9$.

We assessed the robustness of the results produced by VERSO STEP $\#1$ on Dataset $\#1$ when different thresholds are employed to call clonal variants. In the analysis presented in the main text the default VF threshold ($\delta = 90\%$) was employed, which resulted in $m = 29$ clonal variants.

Here, we scanned a number of thresholds in the set $\delta \in \{50\%, 60\%, 70\%, 80\%\}$, which resulted in 31, 31, 30 and 30 variants, respectively. We then assessed the robustness of VERSO STEP $\#1$ computing the accuracy: $\frac{\text{TP+TN}}{\text{TP+TN+FP+FN}}$ by considering as ground-truth the topology returned by VERSO with $\delta = 90\%$. In this case, the true positives (TP) are the rightly inferred edges, the false positives (FP) the wrongly inferred edges, the false negatives (FN) the edges that are present in the ground-truth topology, but are not inferred, the true negatives (TN) the edges that are not inferred and are not present in the ground-truth topology.

As one can see in Figure S7, the accuracy varies between 0.97 and 0.98 in all settings, demonstrating the the results produced by VERSO STEP $\#1$ are robust with regard to the choice of the VF threshold for clonal variant identification.

2.6 Application of VERSO to 1438 samples from RNA sequencing data (Dataset $\#2$)

The application of VERSO to Dataset $\#2$ is discussed in the main text and the output models are displayed in Figure S8 (see Table S3 for further details).

2.7 Expanded clonal variants tree – Dataset $\#2$

In Figure S9 we report the *expanded* clonal variants tree of Dataset $\#2$ (see above).

2.8 Computation time

We assessed the computation time of VERSO STEP $\#1$ in different simulated scenarios, by especially evaluating the impact of variations of the number of samples n. All simulations were performed by employing a specific simulation setting with $m = 14$ clonal variants (simulation id: $\#1$) and we assessed the computation time by varying the number of samples: $n \in \{100, 500, 1000, 5000, 10000\}$. For datasets with more than 1000 samples, random resampling of the original dataset was performed.

For each configuration, we executed 10 independent computations, with 1000 MCMC iterations and 5 restarts, on a single core of a MacBook Pro, processor 2.6 GHz Intel Core i7, 16 GB RAM 2400 MHz DDR4. The distribution of the run times with respect to the distinct configurations is shown in Supplementary Fig. S10.

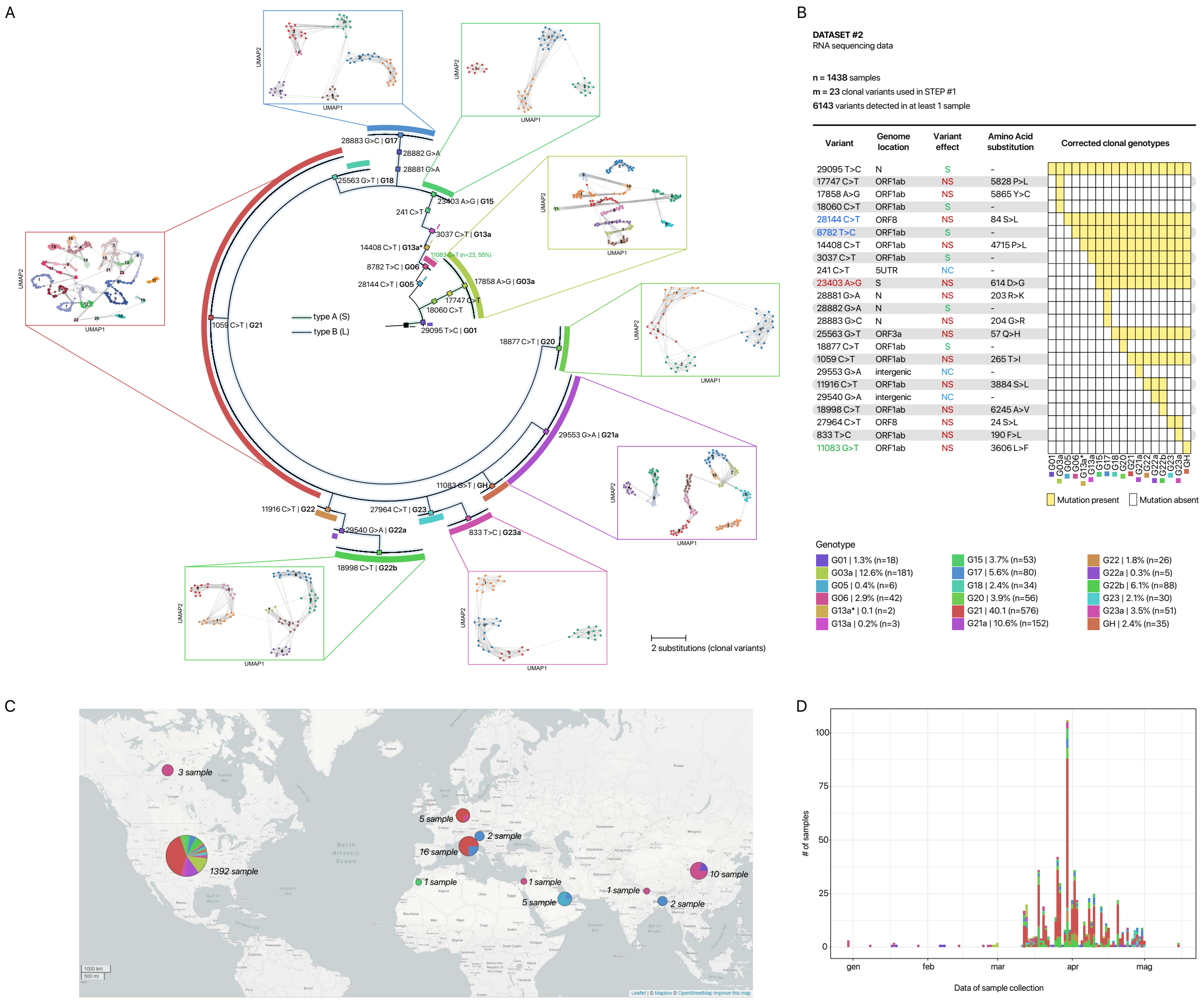

Figure S8: **Viral evolution and intra-host genomic characterization of** 1438 **SARS-CoV-2 samples via VERSO (Dataset #2). (A)** The phylogetic model returned by VERSO STEP #1 from the mutational profile of 1438 samples selected after QC, on 23 clonal variants (VF $> 90\%$) detected in at least 3% of the samples of Dataset #2 (reference genome: SARS-CoV-2-ANC). 18 distinct clonal genotypes are identified by VERSO, 11 of which are identical to those found in the analysis of Dataset #1 and maintain the same label. 6 further clonal genotypes are distinct, but evolutionary consistent and are marked with letters a, $a*$ and b, while clonal genotype GH is associated to homoplastic clonal variant g.11083G>T (the mapping with the lineage nomenclature proposed in [15] is provided in Supplementary File 3). Samples with identical corrected clonal genotypes are grouped in polytomies (visualization via FigTree[16]). Notice that the colors do not correspond with those of Figure 3 of the main text. The green curves juxtaposed to certain polytomies report the number and fraction of samples in which homoplastic mutation g.11083G>T is observed (only if the mutation is detected in at least 10 samples with the same corrected clonal genotype; see Supplementary File 2 for a summary on the samples exhibiting homoplastic clonal variants). The projection of the intra-host genomic composition and similarity computed by VERSO STEP #2 from VF profiles is shown on the UMAP low-dimensional space for the clonal genotypes including ≥ 100 samples. Samples are clustered via Leiden algorithm on the k-nearest neighbor graph ($k = 10$), computed on the Bray–Curtis dissimilarity on VF profiles, after PCA. Solid lines represent the edges of the k-NNG. **(B)** The composition of the corrected clonal genotypes returned by VERSO STEP #1 is shown. Clonal SNVs are annotated with mapping on ORFs, synonymous (S), nonsynonymous (NS) and non-coding (NC) states, and related amino acid substitutions (asterisks label nonsense mutations). Variants colored in blue characterize the B (L) type[17], variant g.23403 A>G (S, p.614 D>G) is colored in red, homoplastic variant g.11083G>T ($ORF1ab$, p.3606L>F) in green. **(C-D)** The geo-temporal localization of the clonal genotypes via Microreact[18] and the prevalence variation in time are displayed.

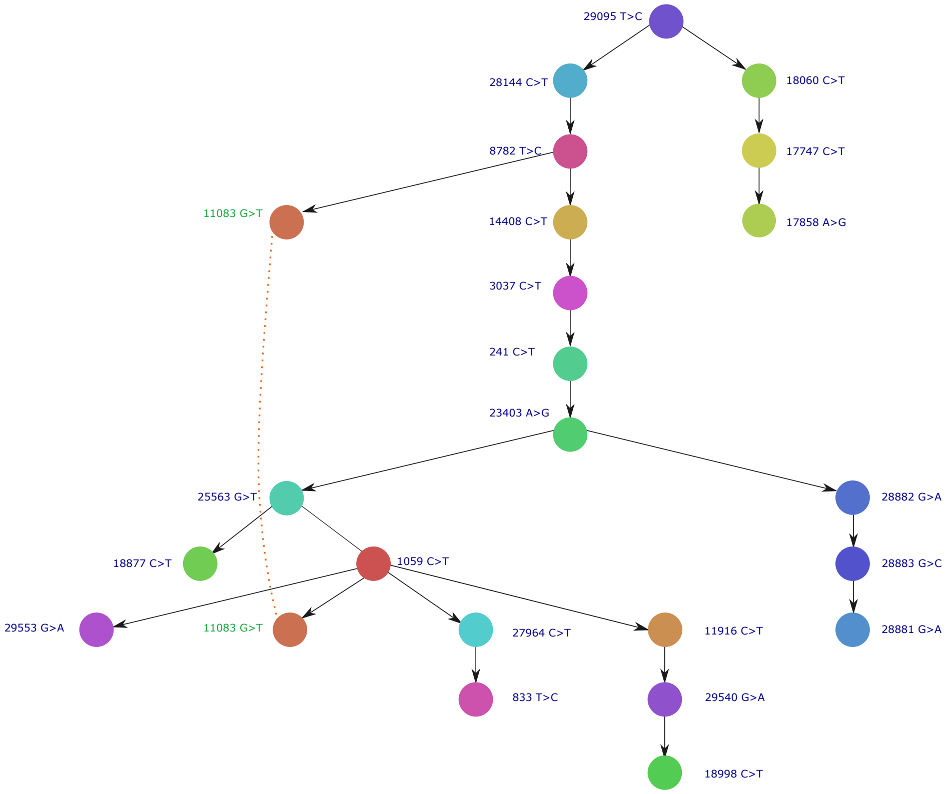

Figure S9: **Expanded clonal variants tree – Dataset #2.** The clonal variants tree **B** representing the inner structure of the output phylogenetic tree inferred from Dataset #2 via VERSO STEP #1 is expanded by duplicating homoplastic variants. Nodes represent clonal variants (consistent colors with Supplementary Figure 8), whereas edges indicate temporal ordering/accumulation relations. Homoplastic mutations were duplicated if observed in at least 10 samples with the same corrected clonal genotype returned by VERSO STEP #1 and are attached after the corresponding characterizing mutation. Homoplastic variants observed in multiple samples with the same corrected clonal genotype are ordered by the number of samples in which they are detected. Dashed lines connect identical homoplastic mutations.

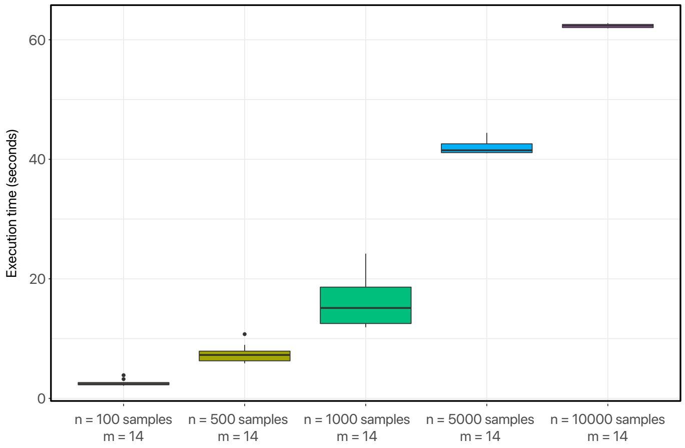

Figure S10: **Computation time**. Computation time of VERSO with respect to the 6 different configurations described in the text; every boxplot displays the distribution of 10 independent computations with 1000 MCMC iterations and 5 restarts. Execution time in seconds.

References

1. Ramazzotti, D., Angaroni, F., Maspero, D., Ascolani, G., Castiglioni, I., Piazza, R., Antoniotti, M. and Graudenzi, A. (2020). Longitudinal cancer evolution from single cells. bioRxiv. https://doi.org/10.1101/2020.01.14.906453.
2. Gusfield, D. (1991). Efficient algorithms for inferring evolutionary trees. Networks 21(1), 19–28. https://doi.org/10.1002/net.3230210104.
3. Kimura, M. (1969). The number of heterozygous nucleotide sites maintained in a finite population due to steady flux of mutations. Genetics 61(4), 893–903. https://www.genetics.org/content/61/4/893.
4. Jahn, K., Kuipers, J. and Beerenwinkel, N. (2016). Tree inference for single-cell data. Genome Biology 17(1), 1. https://doi.org/10.1186/s13059-016-0936-x.
5. Kuhner, M.K. and Felsenstein, J. (1994). A simulation comparison of phylogeny algorithms under equal and unequal evolutionary rates. Molecular Biology and Evolution 11(3), 459–468. https://doi.org/10.1093/oxfordjournals.molbev.a040126.
6. Steel, M.A. and Penny, D. (1993). Distributions of tree comparison metrics—some new results. Systematic Biology 42(2), 126–141. https://doi.org/10.1093/sysbio/42.2.126.
7. Kelleher, J., Etheridge, A.M. and McVean, G. (2016). Efficient coalescent simulation and genealogical analysis for large sample sizes. PLOS Computational Biology 12, 1–22. https://doi.org/10.1371/journal.pcbi.1004842.
8. Hadfield, J., Megill, C., Bell, S.M., Huddleston, J., Potter, B., Callender, C., Sagulenko, P., Bedford, T. and Neher, R.A. (2018). Nextstrain: real-time tracking of pathogen evolution. Bioinformatics 34(23), 4121–4123. https://doi.org/10.1093/bioinformatics/bty407.
9. Skála, Z. and Zrzavỳ, J. (1994). Phylogenetic reticulations and cladistics: Discussion of methodological concepts. Cladistics 10(3), 305–313. https://doi.org/10.1111/j.1096-0031.1994.tb00180.x.
10. Bandelt, H.J., Quintana-Murci, L., Salas, A. and Macaulay, V. (2002). The fingerprint of phantom mutations in mitochondrial DNA data. The American Journal of Human Genetics 71(5), 1150–1160. https://doi.org/10.1086/344397.
11. Soares, P., Ermini, L., Thomson, N., Mormina, M., Rito, T., Röhl, A., Salas, A., Oppenheimer, S., Macaulay, V. and Richards, M.B. (2009). Correcting for purifying selection: an improved human mitochondrial molecular clock. The American Journal of Human Genetics 84(6), 740–759. https://doi.org/10.1016/j.ajhg.2009.05.001.
12. Monsion, B., Froissart, R., Michalakis, Y. and Blanc, S. (2008). Large bottleneck size in cauliflower mosaic virus populations during host plant colonization. PLOS Pathogens 4(10), 1–7. https://doi.org/10.1371/journal.ppat.1000174.
13. Lequime, S., Fontaine, A., Ar Gouilh, M., Moltini-Conclois, I. and Lambrechts, L. (2016). Genetic drift, purifying selection and vector genotype shape dengue virus intra-host genetic diversity in mosquitoes. PLOS Genetics 12(6), 1–24. https://doi.org/10.1371/journal.pgen.1006111.
14. Popa, A., Genger, J.W., Nicholson, M.D., Penz, T., Schmid, D., Aberle, S.W., Agerer, B., Lercher, A., Endler, L., Colaço, H. et al. (2020). Genomic epidemiology of superspreading events in Austria reveals mutational dynamics and transmission properties of SARS-CoV-2. Science Translational Medicine 12(573). https://doi.org/10.1126/scitranslmed.abe2555.
15. Rambaut, A., Holmes, E.C., O'Toole, Á., Hill, V., McCrone, J.T., Ruis, C., du Plessis, L. and Pybus, O.G. (2020). A dynamic nomenclature proposal for SARS-CoV-2 lineages to assist genomic epidemiology. Nature Microbiology 5(11), 1403–1407. https://doi.org/10.1038/s41564-020-0770-5.
16. Rambaut, A. (2009). Figtree v1. 3.1. http://tree. bio. ed. ac. uk/software/figtree/ .
17. Forster, P., Forster, L., Renfrew, C. and Forster, M. (2020). Phylogenetic network analysis of SARS-CoV-2 genomes. Proceedings of the National Academy of Sciences 117(17), 9241–9243. https://doi.org/10.1073/pnas.2004999117.
18. Argimón, S., Abudahab, K., Goater, R.J., Fedosejev, A., Bhai, J., Glasner, C., Feil, E.J., Holden, M.T., Yeats, C.A., Grundmann, H. et al. (2016). Microreact: visualizing and sharing data for genomic epidemiology and phylogeography. Microbial Genomics 2(11), e000093. https://doi.org/10.1099/mgen.0.000093.
